# Defect dipole stretching enables ultrahigh electrostrain

**DOI:** 10.1126/sciadv.adn2829

**Published:** 2024-07-10

**Authors:** Shuo Tian, Binquan Wang, Bin Li, Yiping Guo, Shujun Zhang, Yejing Dai

**Affiliations:** ^1^ School of Materials, Sun Yat-sen University, Shenzhen 518107, P. R. China.; ^2^ State Key Laboratory of Metal Matrix Composites, School of Materials Science and Engineering, Shanghai Jiao Tong University, Shanghai 200240, P. R. China.; ^3^ Institute for Superconducting and Electronic Materials, Faculty of Engineering and Information Sciences, University of Wollongong, Wollongong, NSW 2500, Australia.

## Abstract

Piezoelectric actuators have been extensively utilized as micro-displacement devices because of their advantages of large output displacement, high sensitivity, and immunity to electromagnetic interference. Here, we propose a straightforward approach to design <110>-oriented defect dipoles by introducing A-site vacancies and oxygen vacancies in (K_0.48_Na_0.52_)_0.99_NbO_2.995_ ceramics. As a result, we achieve ultrahigh electrostrains of 0.7% at 20 kV cm^−1^ (with an effective piezoelectric strain coefficient *d*_33_^*^ = 3500 pm V^−1^), outperforming the performance of existing piezoelectric ceramics at the same driving field. The exceptional electrostrain is primarily attributed to the large stretching of defect dipoles when subjected to an applied electric field, a phenomenon that has been experimentally confirmed. Moreover, the strong interaction between these defect dipoles and <110> spontaneous polarizations plays a critical role in minimizing hysteresis and ensuring excellent fatigue resistance. Our findings present a practical and effective strategy for developing high-performance piezoelectric materials tailored for advanced actuator applications.

## Teaser

A giant electrostrain in piezoelectrics is obtained via defect dipole design.

## INTRODUCTION

Piezoelectric actuators have advantages such as large output displacement, high sensitivity, and anti-electromagnetic interference, and have been extensively utilized as a kind of micro-displacement device with great development potential (*1*–*3*). Piezoelectric material is the core component of the piezoelectric actuator, and its electric field–induced strain (electrostrain) behavior stands as a pivotal factor shaping the actuator’s performance. Among the key parameters profoundly impacting piezoelectric actuators are the unipolar electrostrain and the driving electric field (*4*). Notably, a substantial electrostrain value achieved at a low driving electric field is essential for achieving a high effective large-signal piezoelectric strain coefficient (denoted as *d*_33_^*^ = *S*_max_/*E*_max_, with *S*_max_ and *E*_max_ representing the maximum strain and the corresponding electric field, respectively). This condition proves conducive to the practical utilization of piezoelectric actuators (*5*). Nevertheless, attaining both an ultrahigh electrostrain and a substantial effective piezoelectric strain coefficient in piezoelectric materials, especially when considering the generally accepted assessment standard for piezoelectric actuator materials, which involves a low electric field of 20 kV cm^−1^, continues to pose a formidable challenge. In addition, there exists a pressing demand for high-performance lead-free systems to replace the currently prevalent lead-based ferroelectric ceramics, driven by environmental regulations. (K,Na)NbO_3_ (KNN)–based ceramics stand out as among the most promising candidates for lead-free ceramics due to their high Curie temperature and commendable piezoelectric performance. Nonetheless, the relatively modest electrostrain (<0.3%) limits their usage in actuation applications requiring substantial displacement.

In recent years, the approach of designing composition through defect engineering has emerged as an effective strategy to improve the electrostrain performance of perovskite-structure piezoelectric materials (*6*–*14*). It is generally believed that positively and negatively charged point defects tend to form defect dipoles within a unit cell during an aging process. Through the subsequent poling process, the defect dipoles can be reoriented towards the poling direction (*8*, *11*, *12*), which will lead to a macroscopic asymmetry in performance. For instance, in (Bi_0.5_Na_0.5_)TiO_3_ (BNT)–based piezoelectric ceramics, a high asymmetric electrostrain of 2.3% was obtained at a temperature of 220°C by using atomic-scale defect manipulation and mesoscale domain engineering (*10*). A notable unipolar strain of 1.12% (*d*_33_^*^ =1120 pm V^−1^) was achieved in BNT-based ceramics by combining defect dipoles, a reversible relaxor-to-ferroelectric phase transition, and a morphotropic phase boundary (*12*). For the KNN-based system, by forming ($V_{A}^{\prime }-V_{O}^{\cdot \cdot }$) defect dipoles, high bipolar electrostrains of 1.05% and 1.35% under 50 kV cm^−1^ at room temperature, attributed to the synergistic effects of the aligned defect dipoles, multiphase coexistence, and ferroelectricity, were reported for polycrystalline and textured ceramics, respectively, whereas the unipolar electrostrain values are on the order of 0.7% and 0.8%, respectively (*13*, *14*). Previous reports on large electrostrains have predominantly relied on the synergistic effects of defect dipoles with multiphase coexistence, domain engineering, or field-induced phase transition, which make it challenging to experimentally distinguish the contribution of the defect dipoles to the electrostrain, adding to the intriguing nature of this research area. Therefore, there is a pressing need for more comprehensive research to explore the potential of defect dipoles in enhancing electrostrain.

In experimental settings, researchers use different sintering approaches and atmospheres, and they may also regulate the duration of the sintering process, which will greatly tailor the defect concentrations and thus the formation of defect dipoles in perovskite materials. Here, our primary objective is to design <110>-oriented ($V_{A}^{\prime }-V_{O}^{\cdot \cdot }$) defect dipoles by introducing A-site vacancies and oxygen vacancies leveraging a simple nonstoichiometric composition approach, i.e., (K_0.48_Na_0.52_)_(1−*x*)_NbO_(3−*x*/2)_ (*x* = 0.005, 0.01, and 0.015, abbreviated as KNN99.5, KNN99, and KNN98.5, respectively) with a conventional high-temperature solid-state sintering method. During the sintering process, the samples were contained within hermetically sealed crucibles to mitigate the volatilization of the alkali metal elements. As a result, the generation of A-site vacancies was solely facilitated by the deliberate nonstoichiometric composition design, and the vacancies from volatilization are negligible. Through this approach, we successfully achieved defect dipoles with orientation aligned along the <110>-oriented spontaneous polarizations of the KNN ceramics with an orthorhombic phase, which facilitates a robust interaction between defect dipoles and spontaneous polarizations. This approach differs from the conventional B-site acceptor doping strategy, for example, the formation of $\left(M_{B}^{\prime \prime }-V_{O}^{\cdot \cdot }\right)$ defect dipole, which is typically oriented along the <001> direction. This alignment results in a substantial enhancement of the electrostrain response, coupled with reduced strain hysteresis and enhanced stability. It is important to highlight that although the volatilization of A-site elements in KNN100 ceramics is inevitable, we believe that the concentration of defects formed under meticulously controlled sintering conditions and with judicious selection of source materials remains insufficient for the creation of impactful defect dipoles. This inadequacy, in our view, leads to their negligible contribution to the electrostrain, as substantiated by the symmetric strain-field curve (which will be discussed in detail later). Here, we carefully maintain consistent sintering conditions to ensure that the sintering process has a minimal impact on defect dipole formation, notwithstanding the slight deviation from the designed compositions caused by the possible volatilization of the alkali metal elements. The detailed unipolar strain and dielectric properties of (K_0.48_Na_0.52_)_(1−*x*)_NbO_(3−*x*/2)_ ceramics are given in fig. S1. Further analysis is focused on (K_0.48_Na_0.52_)_0.99_NbO_2.995_ (KNN99) ceramics, which have the maximum unipolar strain value. It is worth noting that this composition maintains a purely orthorhombic phase near room temperature and remains free from phase transition under the applied electric field. This characteristic helps mitigate the influence of other factors on the electrostrain. These ceramics demonstrate excellent performance even under a small driving electric field, with a unipolar strain of 0.7% at 20 kV cm^−1^ and an effective piezoelectric strain coefficient *d*_33_^*^ of 3500 pm V^−1^, outperforming all existing state-of-the-art piezoelectric ceramics. Moreover, KNN99 ceramics exhibit several advantages, including low hysteresis, good fatigue resistance, and thermal stability, making them highly promising candidates for piezoelectric actuator applications.

## RESULTS

### Working mechanism of defect dipoles

**Fig. 1.**
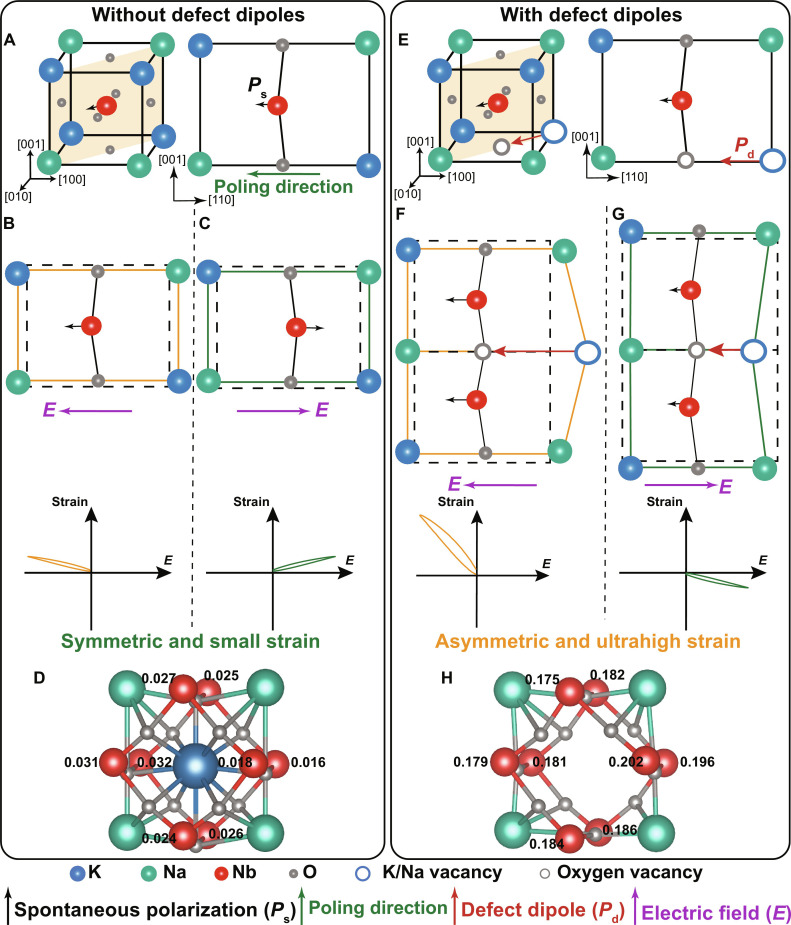
Mechanism of defect dipole-induced ultrahigh electrostrain. (**A**) Schematic diagram of spontaneous polarization along the <110> direction for KNN100 ceramics without defect dipoles. Typical symmetric unipolar electrostrain behaviors under (**B**) negative and (**C**) positive electric fields. (**D**) Ion displacement (Å) of Nb in the model without defect dipoles after applying an electric field of 50 kV cm^−1^ based on the DFT results. (**E**) Schematic diagram of spontaneous polarization and ($V_{A}^{\prime }-V_{O}^{\cdot \cdot }$) defect dipole along the <110> direction for KNN99 ceramics with defect dipoles after the poling process. Asymmetric unipolar electrostrain behaviors under (**F**) negative and (**G**) positive electric fields. (**H**) Ion displacement (Å) of Nb in the model with defect dipoles after applying an electric field of 50 kV cm^−1^ based on the DFT results, in which we take the $\left(V_{K}^{\prime }-V_{O}^{\cdot \cdot }\right)$ defect dipoles as an example.

To clearly demonstrate the working mechanism of defect dipoles, Fig. 1 illustrates the comparison of unipolar electrostrain between (K_0.48_Na_0.52_)NbO_3_ (KNN100) ceramics lacking defect dipoles and KNN99 ceramics containing defect dipoles. Various electric field directions are considered in this schematic. KNN100 ceramics lacking defect dipoles possess an orthorhombic phase of perovskite structure with spontaneous polarizations along the <110> direction (Fig. 1A). As shown in Fig. 1 (B and C), akin to the typical ferroelectric materials, KNN100 displays a relatively small electrostrain value (less than 0.3%) with low hysteresis (*4*, *15*–*17*). This strain arises from the extension or rotation of the spontaneous polarizations when subjected to an electric field. The limited value is due to the confined movement of B-site cations within a small space of oxygen octahedra, which restricts their displacement distance (the polarizability of the spontaneous polarization in the KNN unit cell is about ~10^−40^ F·m^2^) (*8*). In addition, the observed electrostrain values under applying electric fields in opposite directions are basically equal and consistently positive, again, following the behavior observed in a typical ferroelectric material.

Conversely, in the case of KNN99 ceramics with defect dipoles, a distinct scenario emerges. The <110>-oriented ($V_{A}^{\prime }-V_{O}^{\cdot \cdot }$) defect dipoles are able to establish and realign themselves close to the poling direction during the poling process (Fig. 1E, and see note S1 for more details) (*11*). Subsequently, upon the removal of the applied electric field, the alignment of the defect dipoles can induce different strain responses under unipolar electric fields in opposite directions. As shown in Fig. 1F, when the applied electric field direction is the same as the poling direction, the defect dipoles can be greatly stretched due to their strong polarizability [the polarizability of the defect dipole $\left(V_{A}^{\prime }-V_{O}^{\cdot \cdot }\right)$ is about ~10^−37^ F·m^2^, being three orders of magnitude higher than that of the spontaneous polarization] (*8*). This stretching is accompanied by extensive distortion in the lattice of surrounding unit cells, thereby contributing to an exceptional electrostrain. On the basis of the density functional theory (DFT) results (see Materials and Methods for more details), after applying an electric field of 50 kV cm^−1^, the average displacements (0.204 Å for O^2−^ ion, 0.186 Å for Nb^5+^ ion, and 0.191 Å for Na^+^ ion) within KNN99 ceramics far exceed those (0.026 Å for O^2−^ ion, 0.025 Å for Nb^5+^ ion, and 0.014 Å for Na^+^ ion) in KNN100 ceramics (table S1 and Fig. 1, D and H). This disparity highlights that KNN99 ceramics provide a more expansive scope for accommodating lattice distortion.

The reorientation of the defect dipole involves an energy-consuming diffusion process, which requires the migration of ions or vacancies, and thus it becomes challenging to switch the defect dipoles at a relatively low applied electric field (*7*, *18*). Therefore, the defect dipoles will be compressed when subjected to a positive electric field, which leads to a negative unipolar strain (Fig. 1G). It is worth noting that if the interaction between defect dipoles and spontaneous polarizations is strong enough, most spontaneous polarizations and defect dipoles will be maintained in the direction after poling and will not be switched by the applied external electric field, achieving a relatively large negative strain (compression process). The interaction between defect dipoles and spontaneous polarizations is closely related to the orientation relationship between them. As shown in note S2, the calculated electrostatic energy of the <110>-oriented $\left(V_{A}^{\prime }-V_{O}^{\cdot \cdot }\right)$ defect dipole along <110>-oriented spontaneous polarization is about three times that of the <001>-oriented ($B_{\mathrm{Nb}}^{\prime \prime \prime }-V_{O}^{\cdot \cdot }$) defect dipole within KNN ceramics ($B_{\mathrm{Nb}}^{\prime \prime \prime }$ represents a point defect formed by a bivalent cation as an acceptor to replace the Nb^5+^ ion at the B-site.). Therefore, designing defect dipoles based on the orientation relationship between defect dipoles and spontaneous polarizations has an important effect on the electrostrain behavior, including the magnitude of its hysteresis.

### Electrostrain performance

**Fig. 2.**
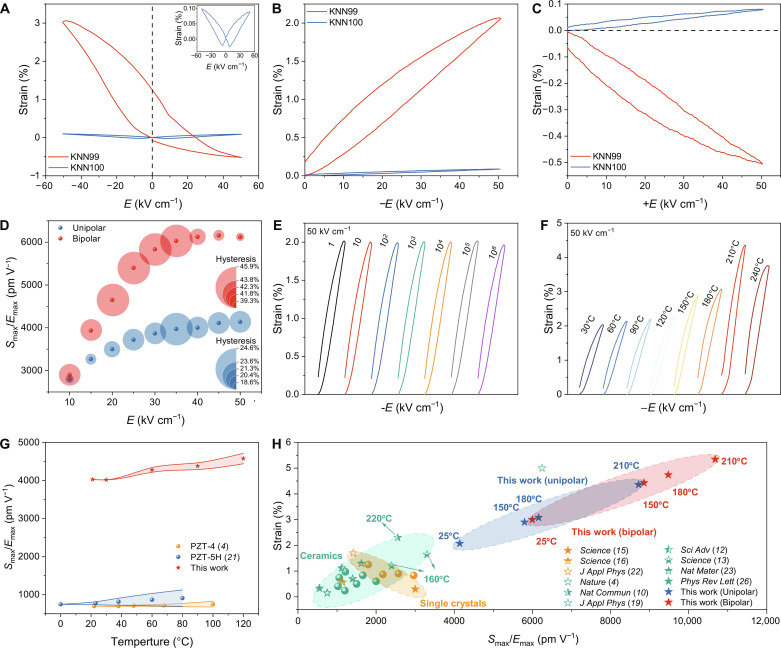
Electrostrain performance of KNN99 ceramics. (**A**) Bipolar *S*-*E* curves, (**B**) unipolar *S*-*E* curves under a negative electric field, and (**C**) unipolar *S*-*E* curves under a positive electric field for KNN99 and KNN100 ceramics (the samples are 0.15 mm in thickness, the applied electric field is at 1 Hz). (**D**) *S*_max_/*E*_max_ and hysteresis under different electric fields calculated according to fig. S6. (**E**) Fatigue test after 10^6^ cycles under −50 kV cm^−1^ at 1 Hz and room temperature; the fatigue frequency is 100 Hz. (**F**) Unipolar electrostrain from 30°C to 240°C. (**G**) Thermal stability comparison of KNN99 ceramics and commercial PZT piezoceramics. The width of the shadow represents the normalized magnitude of the deviation from the room-temperature electrostrain. (**H**) Comparison of the electrostrain performance of KNN99 ceramics with state-of-the-art piezoelectric materials, including lead-based and lead-free ceramics and single crystals.

Figure 2 shows the electrostrain performance of KNN99 ceramics, highlighting the substantial effect of defect dipoles. KNN99 ceramic was subjected to an electric field of 40 kV cm^−1^ and aged for 0.5 hours, and the same treatment was applied to KNN100 ceramic for comparison. In contrast to the nearly symmetric strain–electric field (*S*-*E*) curve observed in KNN100 ceramics, the bipolar electrostrain curve of KNN99 ceramics becomes highly asymmetric, showing a positive electrostrain under the negative electric field and a negative strain under the positive electric field (Fig. 2A). Notably, the bipolar electrostrain under a negative electric field is greatly enhanced, reaching a maximum value on the order of 3.1% at −50 kV cm^−1^, whereas the bipolar electrostrain value of the KNN100 ceramics is only 0.1% at the same electric field. In practical application scenarios, unipolar electrostrain is of great importance for actuators. An ultrahigh unipolar strain of 2.1% with a corresponding effective piezoelectric strain coefficient *d*_33_^*^ of 4200 pm V^−1^ is obtained at room temperature when subjected to an electric field of −50 kV cm^−1^ (Fig. 2B). Moving to Fig. 2C, the unipolar electrostrains of KNN99 and KNN100 ceramics under a positive electric field are also compared. A large unipolar strain of −0.5% under +50 kV cm^−1^ is obtained for KNN99 ceramics, indicating that the aligned defect dipoles exhibit a notable resistance to switching even when exposed to a substantial 50 kV cm^−1^ external electric field. It appears that these defect dipoles engage in robust interactions with spontaneous polarizations. This distinctive behavior contrasts with KNN100 ceramics, which lack defect dipoles and consistently exhibit positive strain under a unipolar electric field (Fig. 2, B and C), demonstrating typical ferroelectric characteristics. Moreover, we conducted real-time monitoring of the deformation exhibited by KNN100 and KNN99 ceramics under a triangle wave voltage of 1000 V and 1 Hz. As illustrated in fig. S2, KNN100 ceramics show a displacement of 0.14 μm (corresponding to a strain of 0.10%) at both positive and negative voltages, while KNN99 ceramics exhibit a negative displacement of −1.63 μm at a positive voltage and a positive displacement of 4.47 μm (corresponding to a strain of 2.98%) at a negative voltage, which is consistent with the aforementioned findings.

Recent findings indicate a remarkable increase in electrostrain simply by reducing the sample thickness (*19*). Akin to earlier observations, the electrostrain of KNN99 ceramics demonstrates a dependence on thickness (fig. S3), escalating from 0.1% with a 1-mm thickness to 2.1% with a 0.15-mm thickness when subjected to an electric field of 50 kV cm^−1^ (it should be noted that all electrostrain values reported in this study are on samples with a thickness of 0.15 mm). In contrast, the electrostrain of BaTiO_3_ (BT) piezoelectric ceramics shows minimal dependence on sample thickness (fig. S3), only rising from 0.13% with a 1-mm thickness to 0.2% with a 0.20-mm thickness. This discrepancy is attributed to the limited creation of defect dipoles in the BT sample, given the nonvolatile nature of cationws (Ba^2+^ and Ti^4+^) during high-temperature sintering in the air. These results further affirm that the asymmetric and substantial electrostrain in KNN99 ceramics originates from defect dipoles within the surface layer, where the surface layer volume ratio increases with reducing the sample thickness. Finite element simulations conducted using COMSOL Multiphysics also indicate that, while the concentration of defect dipoles remains constant, an increase in sample thickness intensifies the clamping effect on the defect dipoles by the ferroelectric domains with higher stiffness around them (see figs. S4 and S5, and refer to note S3 for more details). Consequently, the electrostrain values of KNN99 ceramics exhibit a rapid increase with the thinning of the sample.

The electrostrain characteristics of KNN99 ceramics are thoroughly analyzed in this study, and the results are presented in fig. S6 (A to C). In addition, the electrostrain values, strain hysteresis (=Δ*S*_1/2*E*_max__/*S*_max_, *S*_max_ is the maximum strain, *E*_max_ is the applied maximum electric field, and Δ*S*_1/2*E*max_ represents the electrostrain difference between the increasing and decreasing electric field sweeps at half of *E*_max_), and *d*_33_^*^ under different negative electric fields are calculated and plotted in Fig. 2D, fig. S6D, and table S2. The electrostrain value increases progressively as the electric field increases, with the *d*_33_^*^ increasing from 2800 pm V^−1^ @ −10 kV cm^−1^ to 4200 pm V^−1^ @ −50 kV cm^−1^ based on unipolar strain. These findings indicate the excellent effective piezoelectric response under different electric fields. An essential feature of the unipolar electrostrains is the satisfactory strain hysteresis, measuring less than 25%, comparable to or even lower than the commercial “soft” Pb(Zr,Ti)O_3_ (PZT) systems with hysteresis levels typically >30% (*4*). Furthermore, the robust stability of the defect dipoles contributes notably to the fatigue resistance of KNN99 (Fig. 2E and fig. S7A), and the unipolar strain only experiences a minimal degradation of 5% after 10^6^ cycles. In addition, it is observed that the electrostrain exhibits frequency dependence, with the values diminishing at higher frequencies. Specifically, the electrostrain is on the order of 2.1% at 1 Hz and decreases to 1.5% at 100 Hz at −50 kV cm^−1^ (fig. S8). The observed frequency-dependent behavior observed in KNN99 ceramics (fig. S8C) is attributed to the relaxation time of the defect dipoles responding to the electric field, leading to a more pronounced response at lower frequencies (*20*). To assess the temperature dependence of KNN99 ceramics, comprehensive tests were conducted (Fig. 2F; fig. S7, B and C; and table S3). The results indicate a slight increase in electrostrain with increasing temperature within the lower temperature region, exhibiting good temperature stability from room temperature to 120°C with a variation of less than 14%, comparable with commercial PZT ceramics (Fig. 2G) (*4*, *21*). As the temperature increases further to 210°C, corresponding to the orthorhombic-tetragonal phase transition (*T*_O-T_) (fig. S9), KNN99 ceramics achieve the maximum unipolar electrostrain of 4.4% (*d*_33_^*^ = 8800 pm V^−1^). Of particular importance is that the ceramics showcase excellent fatigue resistance characteristics at elevated temperatures, with variation below 5% after 10^6^ cycles (fig. S10). We also performed the frequency- and temperature-dependent tests to examine permittivity and dielectric loss versus the electric fields for KNN99 and KNN100 ceramics (figs. S11 and S12), both of which show closely aligned values and exhibit similar variation trends. These comparisons serve to rule out the possibility of long-range migration of oxygen vacancies as a mechanism for improving the dielectric properties of KNN99 ceramics. In addition, the electrostrain performance remains remarkably stable even after aging for 12 months (fig. S13), underscoring the excellent long-term stability of the material, which, in turn, indicates that the long-range migration of oxygen vacancies does not occur at room temperature. It is expected, however, that a macroscopic redistribution of defects, specifically involving the long-range migration of oxygen vacancies, may occur under high-temperature treatment, which contributes to the electrostrain.

The unipolar electrostrains and their corresponding *d*_33_^*^ values of KNN99 ceramics are compared to the existing state-of-the-art piezoelectric materials, including lead-based and lead-free piezoelectric ceramics and single crystals [Fig. 2H and table S4 (*4*, *8*, *12*, *13*, *15*, *16*, *19*, *22*–*33*)]. KNN99 ceramics have the highest *d*_33_^*^ value (at 210°C) among all materials studied. For actuator applications with a common driving electric field of 20 kV cm^−1^, KNN99 ceramics also exhibit a substantial unipolar electrostrain of 0.7% and a corresponding *d*_33_^*^ value of 3500 pm V^−1^ with a strain hysteresis of 20% (table S2), far exceeding the performance of commercial PZT-5H ceramics (0.2% @ 20 kV cm^−1^) and other actively studied piezoelectric ceramic systems (*4*, *5*, *13*, *21*). Aside from traditional piezoelectric actuators, defect-engineered KNN ceramics with highly asymmetric positive and negative electrostrains can also provide precise bi-directional driving, which is substantiated by the macro-fiber composite actuators based on the asymmetric strain of defect-engineered KNN ceramics (*34*). This opens up potential applications in a wider range of fields, including robotics, flexible electronics, microfluidic systems, and medical devices.

### Structure features of KNN99 ceramics

To elucidate the contribution of the defect dipole to the ultrahigh electrostrain of KNN99 ceramics, an investigation was carried out, focusing on both macro- and micro-structural features. This comprehensive analysis aimed to eliminate the influence of other contributing factors. The x-ray diffraction (XRD) patterns (fig. S14A) and scanning electron microscopy (SEM) images (fig. S15) indicate that KNN99 ceramics, similar to KNN100 ceramics, maintain an orthorhombic phase with trace impurities resulting from a slight deviation in chemical composition. As a consequence, the coefficient of direct piezoelectric *d*_33_ is reduced to 54 pC N^−1^ versus 96 pC N^−1^ for KNN100 (fig. S14B). Further evidence was obtained through dielectric-temperature curves (fig. S9), in situ temperature-dependent XRD (fig. S16), and in situ temperature-dependent Raman spectra (fig. S17), all of which confirm that the orthorhombic-tetragonal phase transition occurs near 210°C. This finding rules out the possibility of a two-phase coexistence contributing to the room-temperature ultrahigh electrostrain observed in KNN99 ceramics.

Meanwhile, the polarization–electric field (*P*-*E*) loops and the current density–electric field (*J*-*E*) curves are shown in fig. S18, where the low current density of KNN99 ceramics excludes any effect of leakage conductance on the observed electrostrain. In addition, a comparison of the polarization and current density between KNN99 and KNN100 ceramics reveals that the defect dipoles greatly inhibit ferroelectric domain switching under the applied electric field in KNN99 ceramics. Consequently, a relatively slim *P-E* loop with low remnant polarization is observed. These results align consistently with the results of the piezoresponse force microscopy (PFM) characterization (fig. S19). The absence of domain switching behavior from the PFM results further supports the role of defect dipoles in influencing the ferroelectric properties of KNN99 ceramics.

**Fig. 3.**
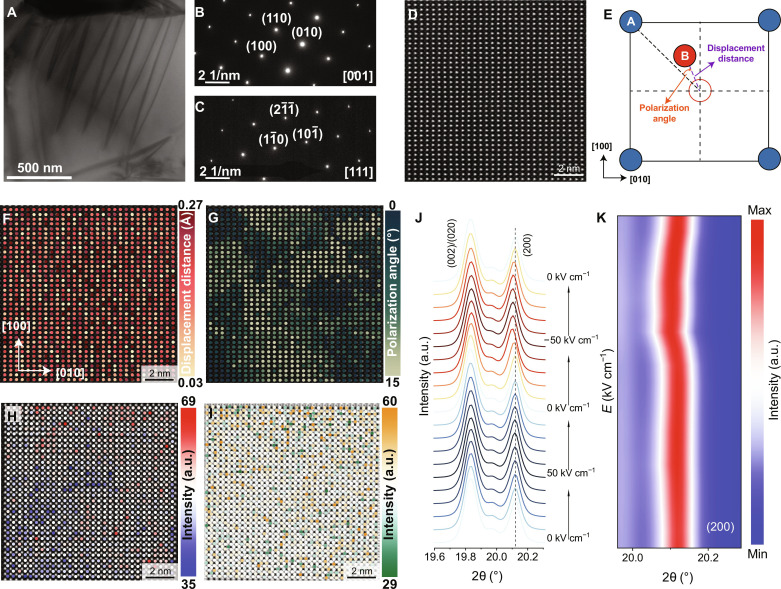
Structural characterization of KNN99 ceramics. (**A**) TEM bright-field image. Selected area electron diffraction (SAED) pattern along (**B**) [001] and (**C**) [111] zone axes. (**D**) HAADF image of KNN99 ceramics. (**E**) Schematic diagram of displacement distance and polarization angle. The distance of the B-site ion deviates from its original position represents the displacement distance. The angle at which the B-site ion deviates from the <110> direction represents the polarization angle. (**F**) Displacement distance and (**G**) polarization angle of B-site ions in KNN99 ceramics based on the HAADF image. Normalized intensities of (**H**) A-site and (**I**) oxygen atom columns. The color of the circles represents the intensity of each atom column. (**J**) In situ SXRD of (002)/(020) and (200) peaks for KNN99 ceramics. (**K**) Contour map of (200) peak intensity as a function of 2θ angle and electric field.

Transmission electron microscopy (TEM) was used to investigate the microstructure of KNN99 ceramics, revealing intriguing observations. Within a representative grain in the bright-field image (Fig. 3A), typical nano-sized wedge-shaped domains with pure orthorhombic symmetry (Fig. 3, B and C, and fig. S20) can be clearly observed. To evaluate the ionic displacement at an atom-scale, aberration-corrected scanning TEM (*C*_s_-corrected STEM) imaging was performed, and the results are given in Fig. 3D. All displacement directions closely align with the <110> direction, which corresponds to the spontaneous polarization direction (fig. S21). Leveraging a custom image recognition script programmed in MATLAB, the location and displacement of the B-site atoms were determined from the high-angle annular dark-field (HAADF) image (Fig. 3E). On the basis of the calculated displacement distance and polarization angle, it was established that all the B-site atoms exhibit displacement along the diagonal directions with a maximum deviation not exceeding 15^°^ (Fig. 3, F and G). The normalized intensities and displacements of the A-site atom in the HAADF image and the oxygen atom columns in an annular bright-field (ABF) image of KNN99 ceramics are demonstrated in Fig. 3 (H and I) and figs. S22 and S23. The drastically varied intensity of the atom columns evidences the existence of A-site vacancies and oxygen vacancies. The increase in vacancy concentration of KNN99 ceramics is further substantiated by the results obtained from electron paramagnetic resonance (EPR) and x-ray photoelectron spectroscopy (XPS) (fig. S24). The elevated vacancy concentration in KNN99 ceramics promotes the formation of defect dipoles, contributing to their unique characteristics and outstanding electrostrain performance. Moreover, an extra peak in the thermally stimulated depolarization current (TSDC) curve observed in KNN99 ceramics confirms the existence of defect dipoles, which would not be dissociated until 330°C, indicating the stability of defect dipoles (fig. S25).

The structural evolution of KNN99 ceramics using in situ synchrotron XRD (SXRD) under different electric fields was investigated to further reveal the origin of ultrahigh unipolar electrostrain, as shown in Fig. 3J and fig. S26. Upon close examination of the enlarged plot of the {200} peaks, two key observations emerge. First, the shape of these peaks remains unchanged as the electric field varies, indicating the absence of any phase transition during field application. Second, the integrated area ratio of the (002) peak to the (200)/(020) peak remains basically constant throughout the electric field variation, revealing that domain wall motion and polarization rotation are not notably affected by the applied electric field. However, with the increase of the negative electric field, the (200) peak gradually shifts to lower angles (Fig. 3K), indicating a lattice expansion (lattice distortion). At the maximum electric field of −50 kV cm^−1^, the 2θ shift angle of the (200) peak for KNN99 ceramics is about 0.012°, corresponding to an electrostrain of 0.06%, primarily arising from the lattice expansion, which is close to the electrostrain value of KNN100 ceramics in Fig. 2B. This implies that the extension of the ferroelectric spontaneous polarizations contributes only marginally to the ultrahigh strain observed in KNN99 ceramics.

The substantial strain observed is a result of the large distortion in the lattice of neighboring unit cells, induced by the stretching of the defect dipole under the applied electric field. As aforementioned, the polarizability of defect dipoles is three orders of magnitude higher than that of ferroelectric spontaneous polarizations, which means that they can be stretched more than the ferroelectric polarizations under the same applied electric field and greatly affect the local strain behavior. It is crucial to recognize that XRD patterns predominantly capture diffraction peaks from the regular crystal planes in the lattice. Given that defect dipoles constitute only a small fraction (approximately 1 mol%) of the material, the distortion of the neighboring lattices they cause has a negligible impact on the shift of diffraction peaks under an electric field. Therefore, while the observed XRD peak shift represents only a minor portion of the overall electrostrain, it fails to discern the primary effect induced by defect dipoles. The contribution of defect dipoles can be corroborated by DFT calculations (see Fig. 1H), where the defect dipole induces a substantial local strain. When subjected to a positive electric field, no detectable shift is observed for the (200) peak, indicating that the negative electrostrain predominantly results from the compression of defect dipoles. As a comparison, the (200) diffraction peaks of the KNN100 ceramics shift to lower angles under both positive and negative electric fields due to the lattice expansion, as shown in fig. S27B. Under an electric field of positive and negative electric fields of 50 kV cm^−1^, the shift angles of the (200) peak basically remain the same value, being 0.007° and 0.008°, respectively, which indicates that the ferroelectric domain can be switched under the applied electric field in KNN100 ceramics, and the electrostrain comes from the switching and extension of ferroelectric domains.

### Theoretical simulation

**Fig. 4.**
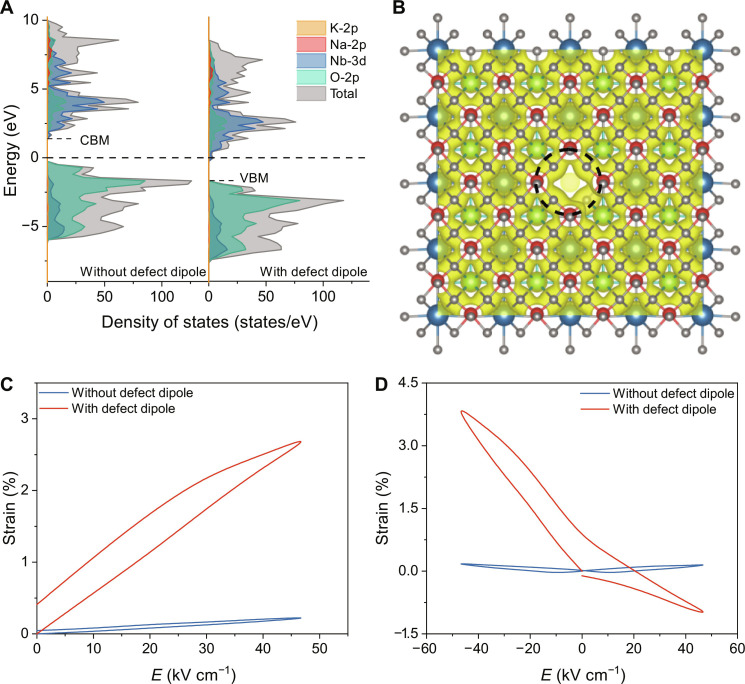
First-principles calculations. (**A**) Total and partial densities of states of KNN ceramics with a defect dipole and without a defect dipole. Fermi level is at 0 eV. (**B**) Charge density of KNN ceramics with a defect dipole. (**C**) Unipolar and (**D**) bipolar electrostrain of KNN ceramics with a defect dipole and without a defect dipole simulated by applying a [110] direction electric field.

We conducted first-principles calculations to further understand the impact of <110>-oriented ($V_{A}^{\prime }-V_{O}^{\cdot \cdot }$) defect dipoles on the electrostrain behavior. We analyzed the specific structural characteristics at a local level using a 4 × 4 × 4 supercell for DFT calculations. Our investigation focused on the relative differences between two types of crystals: those without a defect dipole and those with a defect dipole (fig. S28). For the model with a defect dipole, an adjacent $V_{K}^{\prime }$ and a $V_{O}^{\cdot \cdot }$ are introduced to form a defect dipole in the center of the supercell. Figure 4A shows the total and partial densities of states of KNN ceramics without and with a defect dipole. The presence of oxygen vacancy and A-site vacancy causes a downward shift of the valence band maximum (VBM) and the conduction band minimum (CBM), resulting in a reduced bandgap from 1.53 eV to 1.32 eV (*10*). In addition, the defects in the lattice also cause an uneven distribution of local charge density (Fig. 4B and figs. S29 and S30), consequently inducing a larger polarity within the supercell and enabling larger distortions when subjected to an electric field.

Unipolar and bipolar *S*-*E* curves were also simulated by applying an electric field of −50 kV cm^−1^ along the [110] direction, which is consistent with the spontaneous polarization orientation of KNN ceramics and the ($V_{A}^{\prime }-V_{O}^{\cdot \cdot }$) defect dipole, as shown in Fig. 4 (C and D). For the model without a defect dipole, we only consider the contribution of domain switching, whereas for the model with a defect dipole, the contributions of both the defect dipole and domain are considered. The simulation curves closely resemble the experimental data, yielding a unipolar electrostrain of 2.8%. This value surpasses the experimental results (2.1%), possibly due to the higher defect dipole concentration and their enhanced alignment with the external electric field within the established model. This suggests the potential for further improvements in electrostrain performance through defect dipole optimization.

## DISCUSSION

In summary, we report a straightforward approach to simultaneously achieve ultrahigh electrostrain and effective piezoelectric strain coefficient by compositionally designing <110>-oriented defect dipoles within (K_0.48_Na_0.52_)_0.99_NbO_2.995_ ceramics. Both experimental and simulation results demonstrate that the achieved exceptional electrostrain primarily stems from the stretching of defect dipoles under an applied electric field, highlighting the immense potential of defect dipoles in enhancing the electrostrain response. The combination of the advantages of ultrahigh electrostrain, exceptional effective piezoelectric strain coefficient, low strain hysteresis, excellent fatigue resistance, and good thermal stability makes KNN99 ceramics highly promising lead-free ceramics for piezoelectric actuator applications that demand substantial strain and precision over a broad temperature range.

## MATERIALS AND METHODS

### Sample preparation

The (K_0.48_Na_0.52_)_(1−*x*)_NbO_(3−*x*/2)_ (*x* = 0, 0.005, 0.01, and 0.015) ceramics were fabricated by a conventional high-temperature solid-state sintering method. For the composition with *x* = 0, the samples were sintered in an oxygen atmosphere, and the crucible was made airtight to prevent the K and Na elements from volatilizing. Na_2_CO_3_ (99.8%), K_2_CO_3_ (99.5%), and Nb_2_O_5_ (99.5%) powders were selected as raw materials. The raw materials were mixed and ball-milled with yttrium-stabilized zirconia balls in ethanol for 12 hours. The dried powders were calcined at 850°C for 3 hours in the air, followed by a ball mill for another 12 hours. Then, the calcined powders were uniaxially pressed into pellets with a diameter of 10 mm and a thickness of 1 mm under 200 MPa. Polyvinyl butyral (PVB) was added as a binder and burned off at 500°C after pressing. The samples were sintered in a sealed alumina crucible at 1100°C for 4 hours embedded in the self-source. To prevent the volatilization of elements K and Na, the crucibles were hermetically sealed during the sintering process, while orthorhombic phase Nb_2_O_5_ raw powder was used as the starting material, which will effectively mitigate the evaporation of alkali metal (*35*). Samples were polished, thinned to 0.15 mm, and fired with silver on both sides as electrodes for electrical property measurement. The electrodes were screen-printed to a controlled diameter of 7 mm (38.5 mm^2^) with a thickness of about 10 μm.

### Electrical measurements

To form and align all defect dipoles, (K_0.48_Na_0.52_)_(1−*x*)_NbO_(3−*x*/2)_ ceramics were poled at 40 kV cm^−1^ for 0.5 hours under a negative direct current field and aged for 1 day before electrical measurements. The dielectric-temperature curves were measured by an impedance analyzer (E4990A, Agilent Technologies Inc., CA, USA) with frequencies of 1, 10, and 100 kHz. The *P*-*E* loops, *J*-*E* curves, and *S*-*E* curves were measured by a ferroelectric tester (aix-ACCTF Analyzer 1000, Aachen, Germany) with a high-voltage amplifier (Trek 610E, TREK, USA) at a frequency of 1 Hz. A temperature controller was used to produce in situ thermal field measurements in electrostrain tests. The assessment of fatigue resistance was performed at a frequency of 100 Hz, with subsequent testing carried out at 1 Hz after specific numbers of cycles. The fatigue testing process extended over a duration of 3 hours. The piezoelectric coefficient *d*_33_ was measured by a quasi-static piezoelectric coefficient meter (Institute of Acoustics, Chinese Academy of Sciences, ZJ-6BN). The real-time displacements of the KNN100 and KNN99 ceramics were recorded by a laser-ranging system. A 0.75-V alternating current triangle wave output with a 1-Hz frequency from the waveform generator (DG1022Z, Rigol, China) is amplified 1000 times by the high-voltage amplifier. A mixed-domain oscilloscope (MDO3024, Tektronix Instruments, USA) and a laser scanning vibrometer (LV-S01, Sunny Optical Technology Co. Ltd., China) simultaneously monitor the voltage and real-time displacement of the samples.

### Structure characterizations

The phase structure and in situ variable-temperature XRD of the samples were determined by XRD analysis (D8 ADVANCE, Bruker, Germany) with Cu Kα radiation. The sample surface was etched to 3 nm by Ar ions and then tested by x-ray photoelectron spectroscopy (Escalab 250Xi, Thermo Fisher Scientific, Britain) equipped with a standard monochromatic AlK*a* excitation source (*hv* = 1361 eV). X-band (9.4 GHz) continuous-wave EPR (Bruker ER200-SRC-10/12, Germany) was used to collect the signal of oxygen vacancy. The Raman spectra were measured over the temperature range of 30°C to 210°C with an inVia reflex Raman spectrometer (Renishaw, laser excitation: 532 nm).

The microscopic morphology was observed by SEM (Sigma 300, Zeiss, Germany). The STEM samples were prepared by a focused ion beam, and the bright-field image and selected area electron diffraction (SAED) pattern were acquired on an FEI Talos F200X G2 operating at 200 kV. The HAADF image for A-site atomic projection and the ABF image for oxygen atom projection were performed using a *C_s_*-corrected Titan Cubed Themis G2 microscope operating at 300 kV. The location and displacement of the B-site atoms were determined using a homemade image recognition script programmed in MATLAB. The local poling experiment was carried out on an atomic force microscope (AFM) with the functionality of a piezo-force microscope (Cypher S, Asylum Research, USA) for the polished ceramic sample.

In situ electric field SXRD experiments were performed at beamline 14B1 of the Shanghai Synchrotron Radiation Facility using x-ray with a photon energy of around 18 keV and a wavelength of 0.6887 Å. The Debye diffraction rings were collected on a 2D flat panel detector, which has 3072 × 3072 pixels and a pixel size of 73 × 73 μm^2^. A bipolar electric field of 50 kV cm^−1^ was applied to the sample in steps of 10 kV cm^−1^ parallel to the incident beam direction, with an exposure time of 7 s for each electric field step to ensure that the Debye diffraction ring is at optimum resolution. Before each exposure, the sample was held at a steady voltage for 60 s at each electric field step to ensure sample stability. Fit2D software was utilized here to fit the SXRD peaks.

### First-principles calculations

First-principles calculations based on DFT are performed using VASP (*36*, *37*), where the Perdew-Burke-Ernzerhof generalized gradient approximation method is applied (*38*). The cutoff energy for plane wave basis is set to 450 eV. A Γ-centered Monkhorst-Pack (*39*) 4 × 4 × 1 k-mesh is adopted to sample the Brillouin zone. For electrical transport calculations, the k-mesh is further increased to 7 × 7 × 1. The convergence criteria were set to be 1.0 × 10^−5^ eV and 0.01 eV Å^−1^ per atom. A 4 × 4 × 4 supercell of the (K_0.5_Na_0.5_)NbO_3_ model was presented for the calculation of the electrostrain curve under voltage. In the model featuring a defect dipole, we introduced an adjacent $V_{K}^{\prime }$ and a $V_{O}^{\cdot \cdot }$ to create a defect dipole at the center of the supercell. To maintain a charge-neutral condition, a positive net charge, represented by an A-site vacancy positioned far from the defect dipole, was added to the entire system during calculations using the NEGLECT parameter. To characterize the intrinsic and defect-induced polarizations, we conducted calculations for charge distribution and electronic localization function, as illustrated in fig. S30. In the model without a defect dipole, the electronic densities primarily concentrate around the O atoms, forming ion bonds with alkali metal ions, indicative of intrinsic polarization. Notably, the defect-induced polarization effect becomes more pronounced in the model with a defect dipole, resulting in a more uneven charge distribution around the vacancies. Furthermore, we calculated the strain curve under various electric field intensities, ranging from −50 to 50 kV cm^−1^ with increments of 5 kV cm^−1^ at 20 points. At each point, the lattice parameters were adjusted according to the corresponding voltage. Subsequently, the strain curves, reflecting the electric field intensity, were fitted accordingly. All the systems are fully relaxed before the calculations of electronic structures, in which spin-orbit coupling effects are included.

## Supplementary Materials

### This PDF file includes:

Figs. S1 to S32 Notes S1 to S3 Tables S1 to S4 References
