# Supplementary material for: Defect dipole stretching enables ultrahigh electrostrain
Source: Sci Adv. 2024 Jul 10;10(28):eadn2829. doi: 10.1126/sciadv.adn2829 (PMC11235158; doi:10.1126/sciadv.adn2829)
Supplement: Supplementary file 1 — Figs. S1 to S32 Notes S1 to S3 Tables S1 to S4 References [file sciadv.adn2829_sm.pdf]

Science Advances
AAAS

# Supplementary Materials for

## Defect dipole stretching enables ultrahigh electrostrain

Shuo Tian *et al.*

Corresponding author: Bin Li, libin75@mail.sysu.edu.cn; Yiping Guo, ypguo@sjtu.edu.cn; Shujun Zhang, shujun@uow.edu.au; Yejing Dai, daiyj8@mail.sysu.edu.cn

*Sci. Adv.* **10**, eadn2829 (2024)
DOI: 10.1126/sciadv.adn2829

**This PDF file includes:**

Figs. S1 to S32
Notes S1 to S3
Tables S1 to S4
References

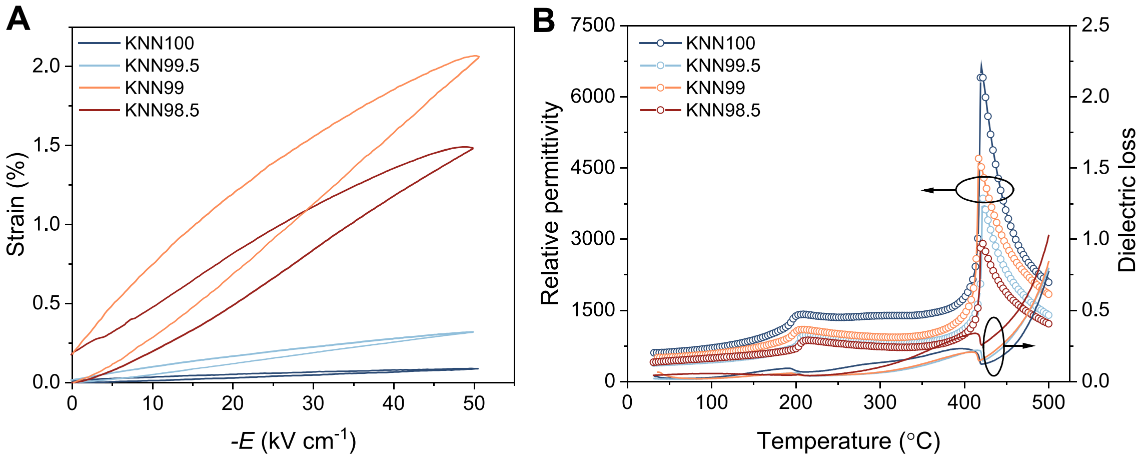


**Fig. S1. Performance of KNN ceramics with different defect concentrations. (A)** Unipolar strains and **(B)** temperature dependent of relative permittivity and dielectric loss at 1 kHz of of $(K_{0.48}Na_{0.52})_{(1-x)}NbO_{(3-x/2)}$ ceramics.

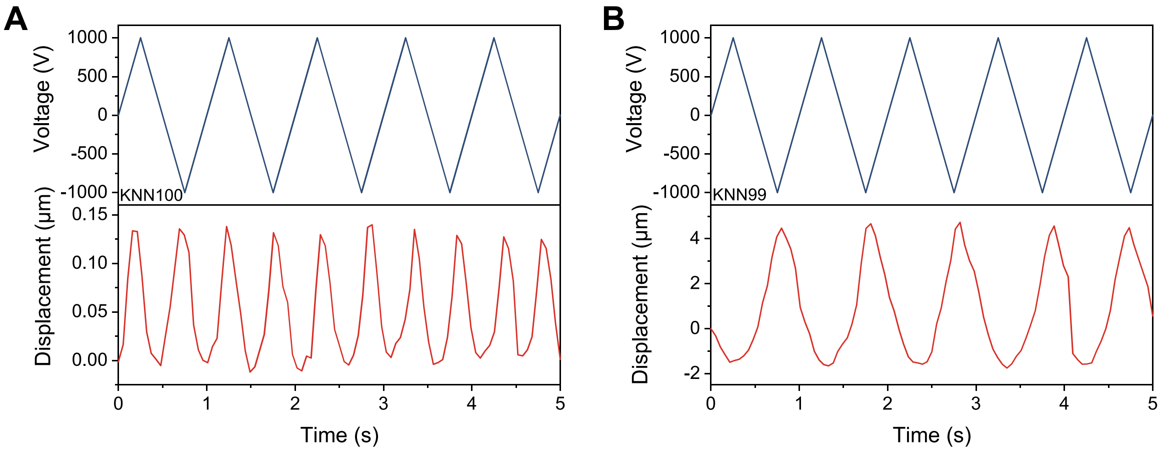


**Fig. S2. Real-time deformation of (A) KNN100 and (B) KNN99 ceramics.**

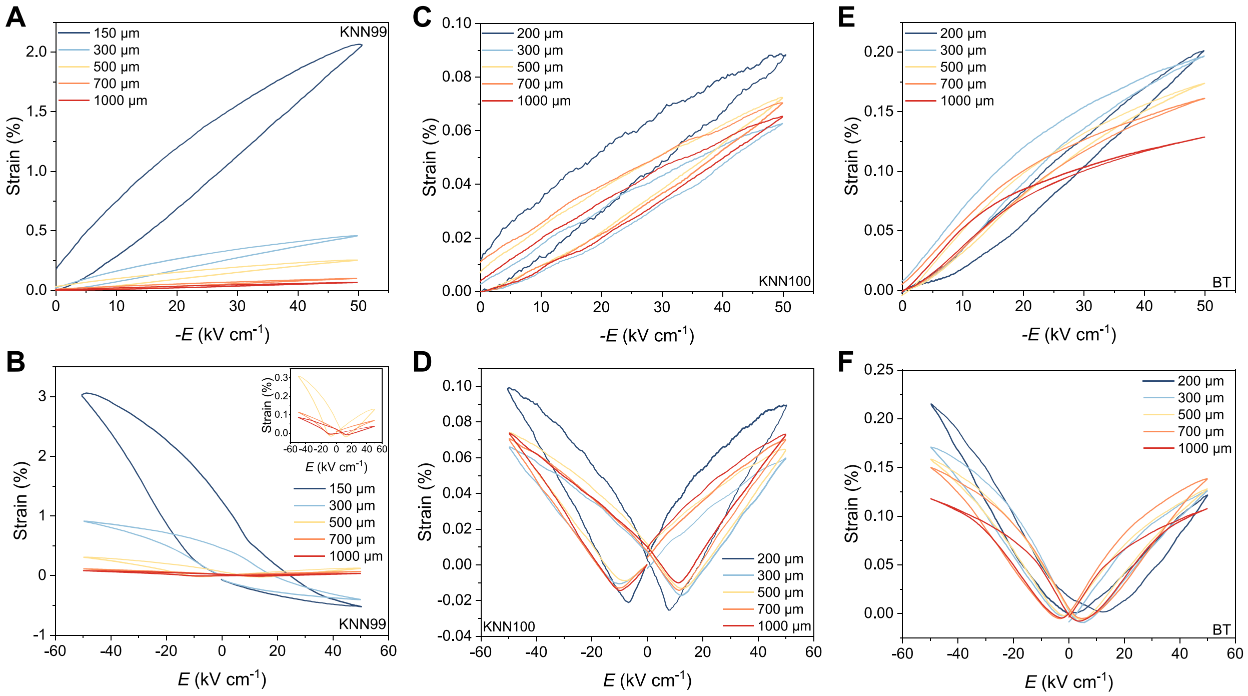


**Fig. S3. Comparison of the electrostrains for KNN99, KNN100, and $BaTiO_3$ ceramics with different thicknesses at room temperature.** (**A**) Unipolar strains and (**B**) bipolar strains of KNN99 ceramics with different thicknesses. (**C**) Unipolar strains and (**D**) bipolar strains of KNN100 ceramics with different thicknesses. (**E**) Unipolar strains and (**F**) bipolar strains of $BaTiO_3$ ceramics with different thicknesses.

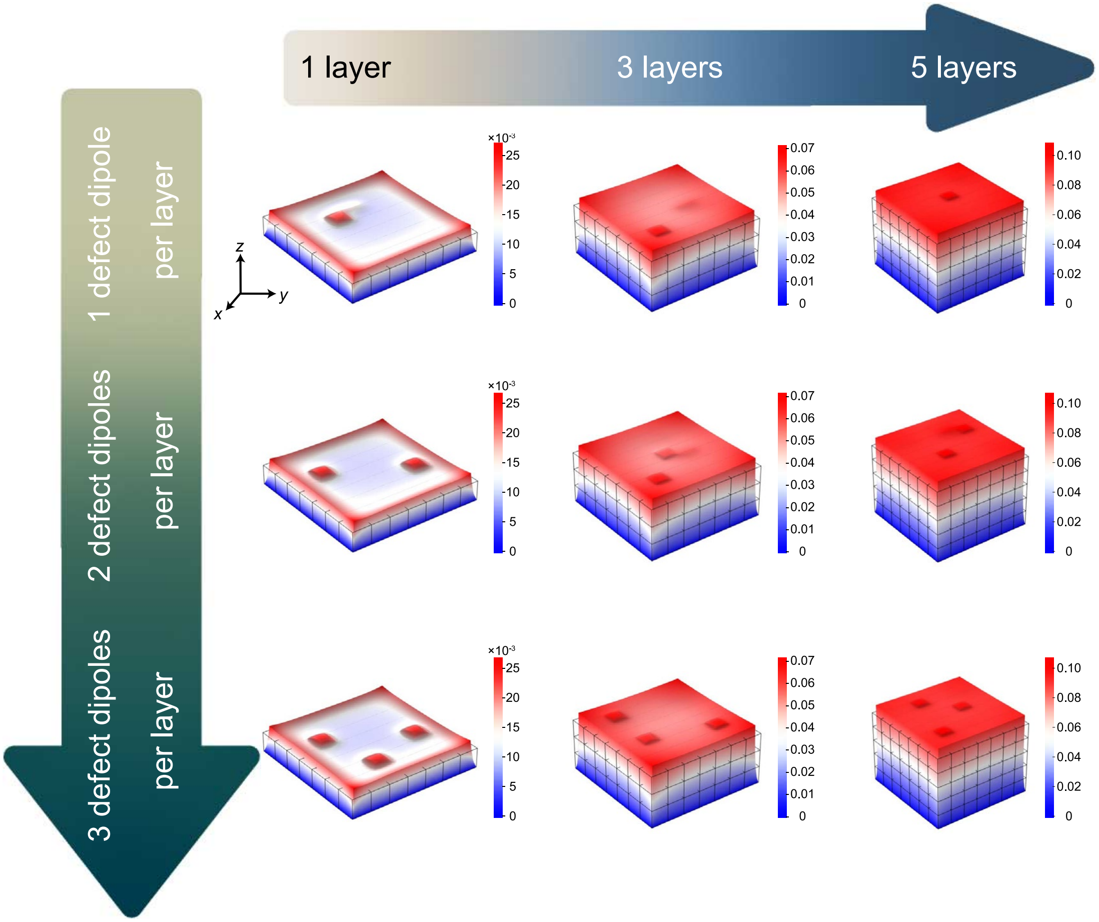


**Fig. S4. Finite element simulations for the impact of thickness on electrostrain in piezoelectric ceramics with aligned defect dipoles.** The scale bar represents the displacement (μm) in the *z*-direction, and the number of layers is related to the thickness of sample.

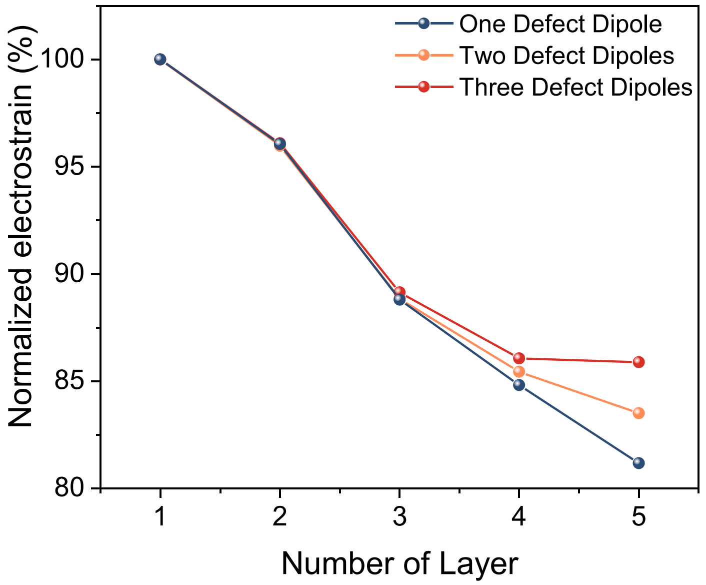


**Fig. S5. The sample thickness dependence of electrostrain in piezoelectric ceramics with aligned defect dipoles, based on finite element simulations.**

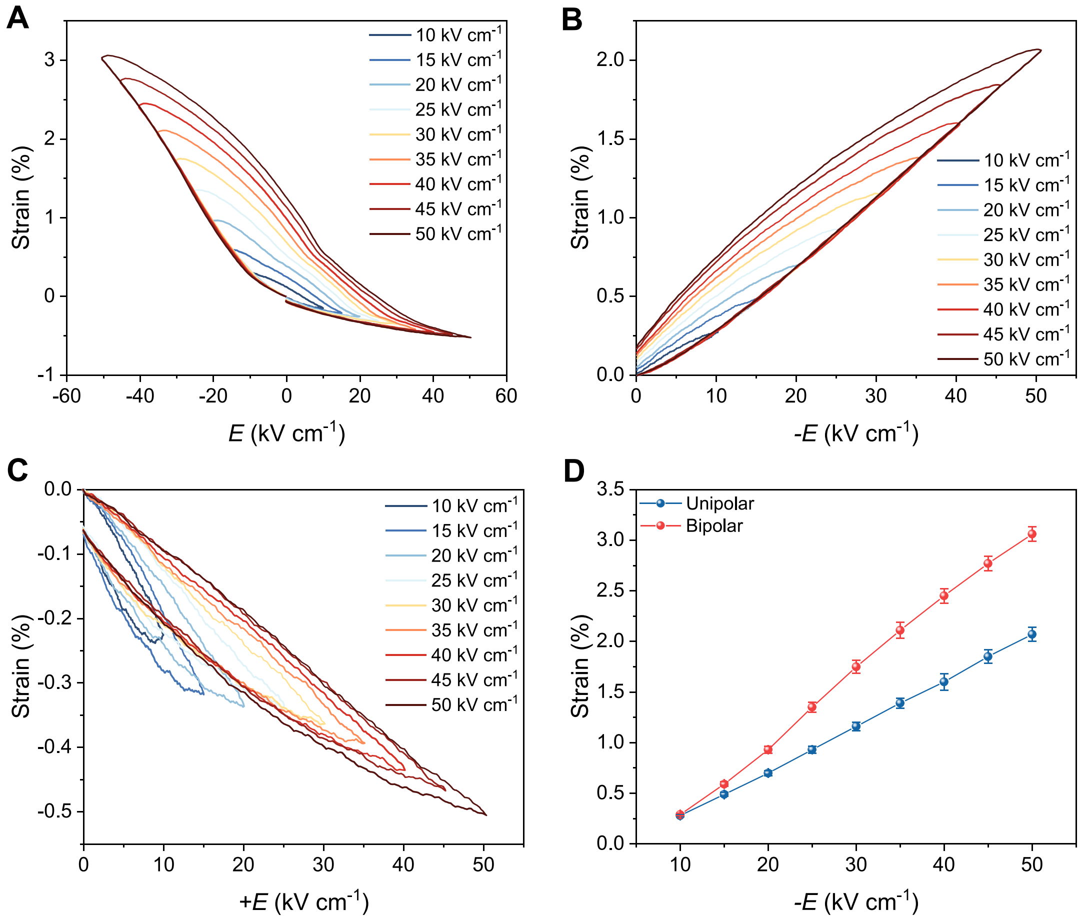


**Fig. S6. Electrostrain performances of KNN99 ceramics under different electric fields.** (**A**) Bipolar *S*-*E* curves. Unipolar *S*-*E* curves under (**B**) negative and (**C**) positive electric fields. (**D**) Unipolar and bipolar strain values of KNN99 ceramics under different electric fields.

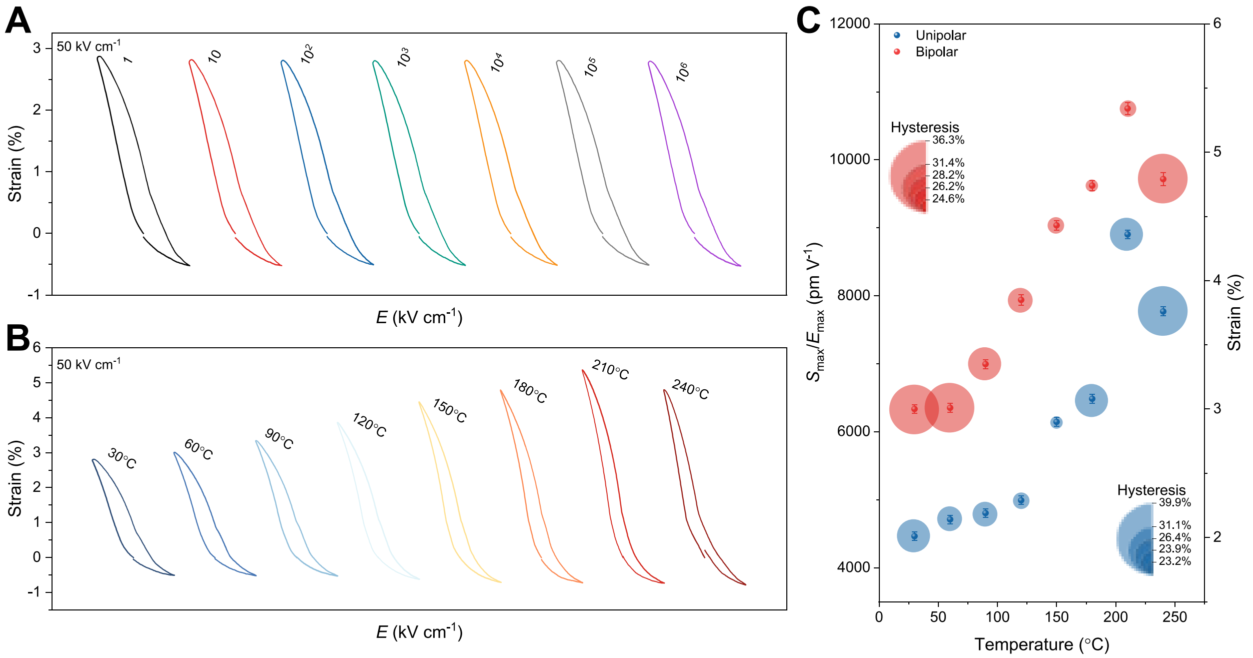


**Fig. S7. Electrostrain performance of KNN99 ceramics.** (**A**) Fatigue tests of bipolar electrostrain under an electric field of 50 kV cm$^{-1}$ (1 Hz). (**B**) Bipolar strain from 30 °C to 240 °C. (**C**) Strain, $S_{max}/E_{max}$, and hysteresis under different temperatures calculated from Fig. 2F and Fig. S6B.

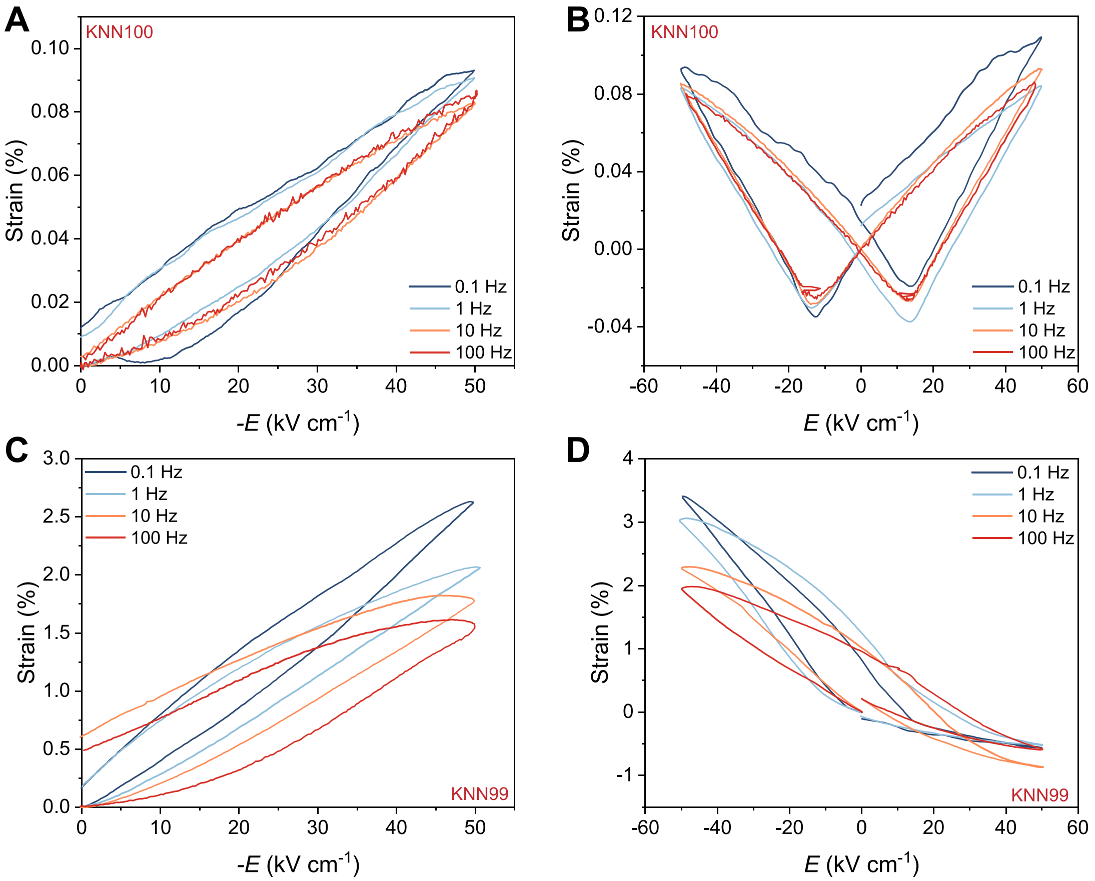


**Fig. S8. Electrostrain performance of KNN100 and KNN99 ceramics under different frequencies.** (**A**) Unipolar strains and (**B**) bipolar strains of KNN100 ceramics. (**C**) Unipolar strains and (**D**) bipolar strains of KNN99 ceramics.

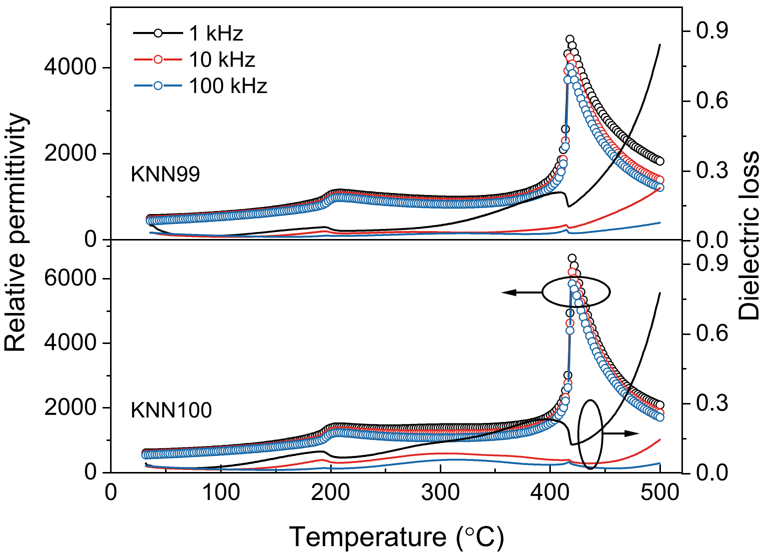


**Fig. S9. Temperature dependent of relative permittivity and dielectric loss of KNN99 and KNN100 ceramics at 1 kHz, 10 kHz, and 100 kHz.**

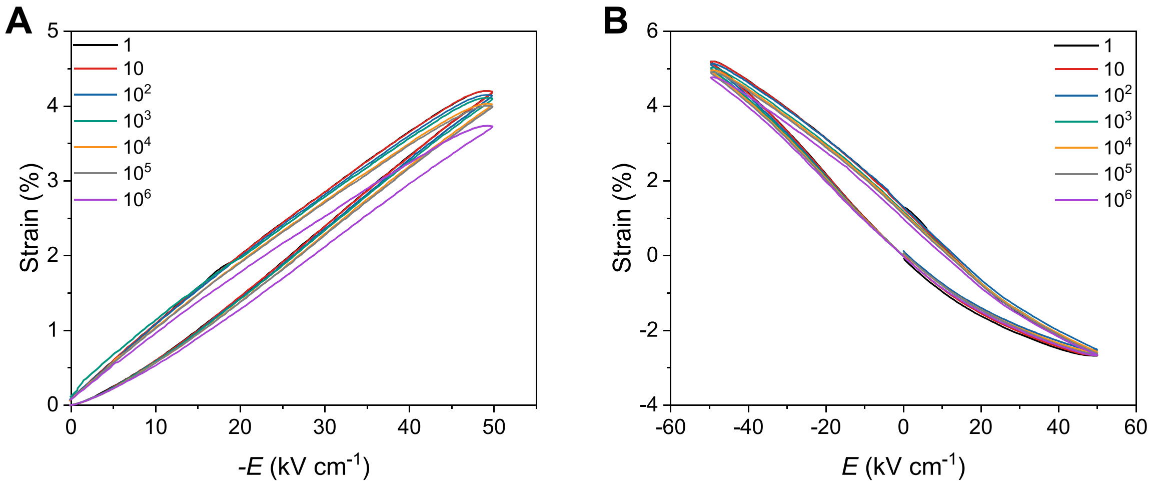


**Fig. S10. Fatigue test of KNN99 ceramics a fter $10^6$ cycles under 50 kV cm$^{-1}$ at 1 Hz and 210 °C. (A)**: Unipolar strains and **(B)**: Bipolar strains.

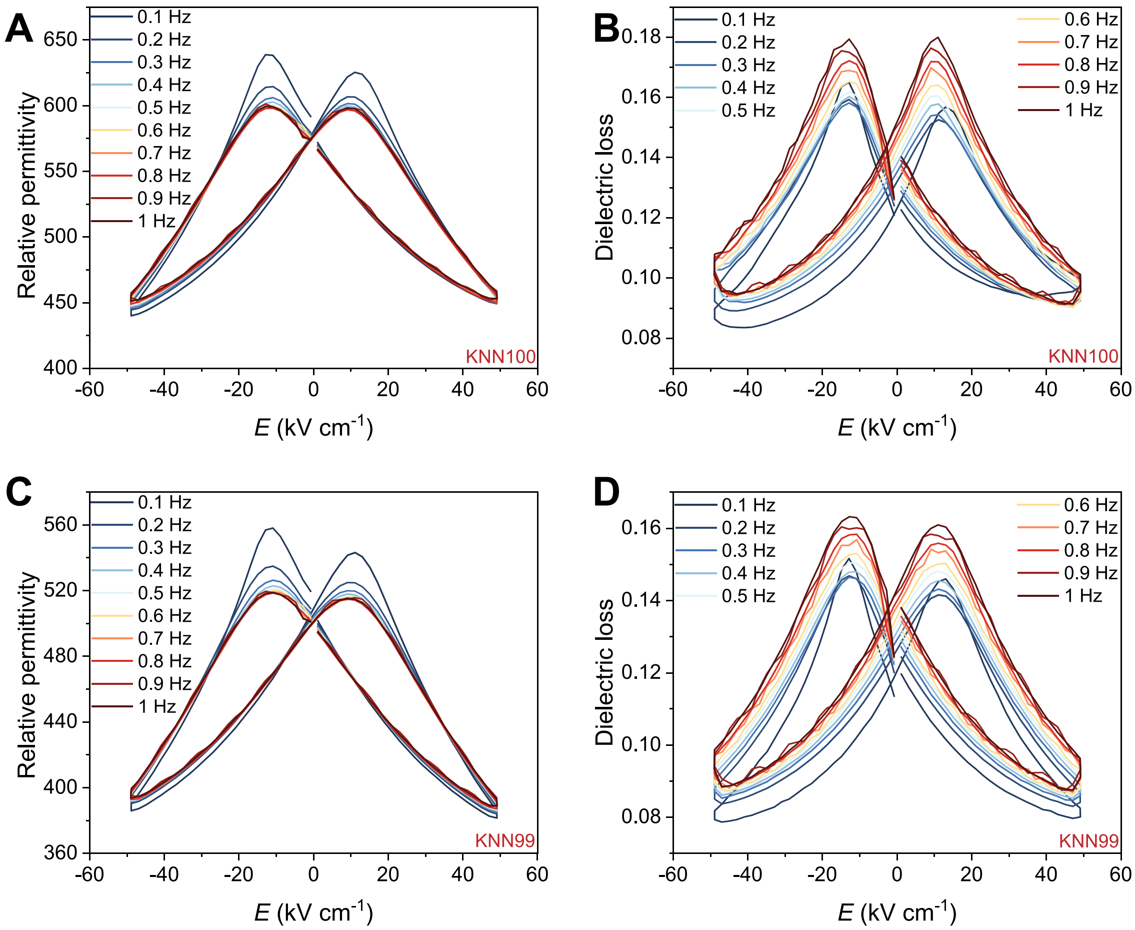


**Fig. S11. Frequency dependence of relative permittivity and dielectric loss versus the electric fields for KNN ceramics.** (**A**, **B**): KNN100 ceramics and (**C**, **D**): KNN99 ceramics.

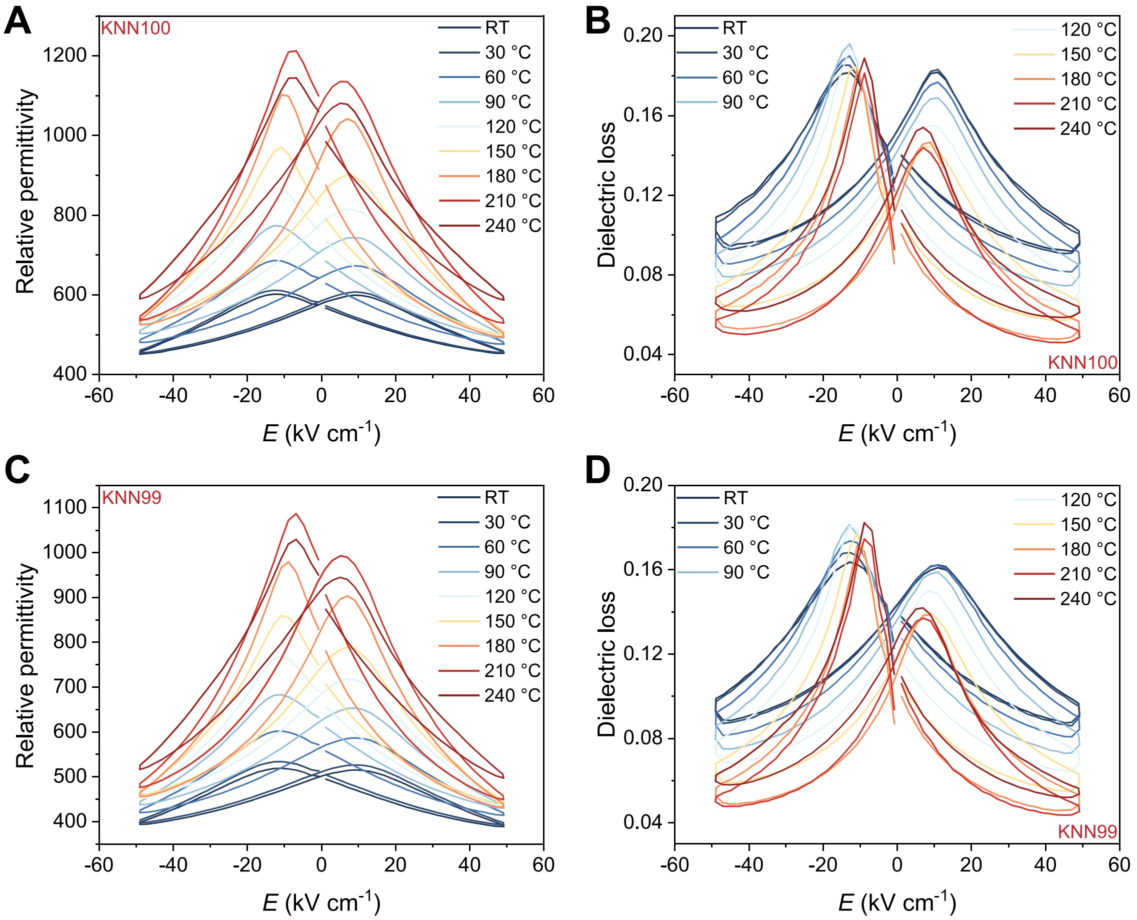


**Fig. S12. Temperature dependence of relative permittivity and dielectric loss versus the electric fields for KNN ceramics.** (**A**, **B**): KNN100 ceramics and (**C**, **D**): KNN99 ceramics.

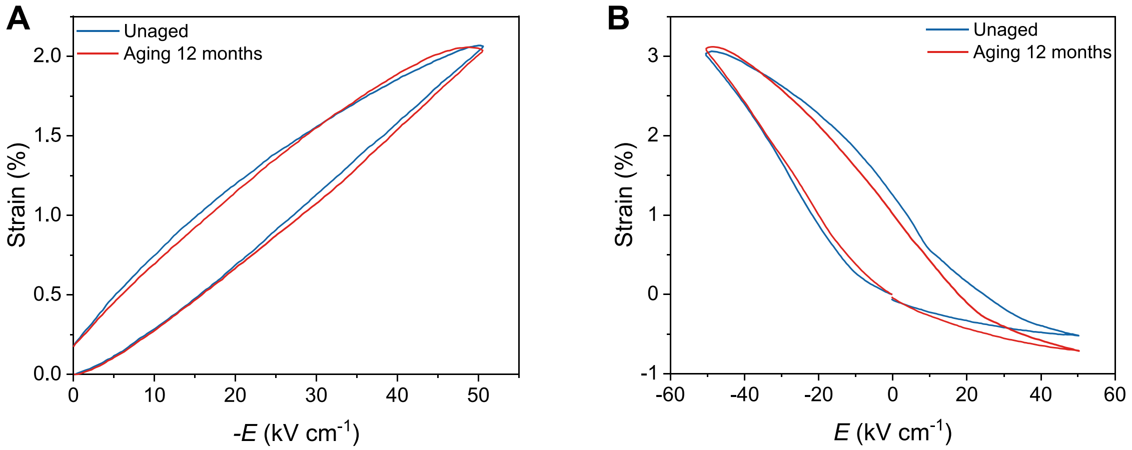


**Fig. S13. *S*-*E* curves of aged KNN99 ceramics.** (**A**) Unipolar and (**B**) bipolar *S*-*E* curves measured for KNN99 ceramics before aging and after aging for 12 months.

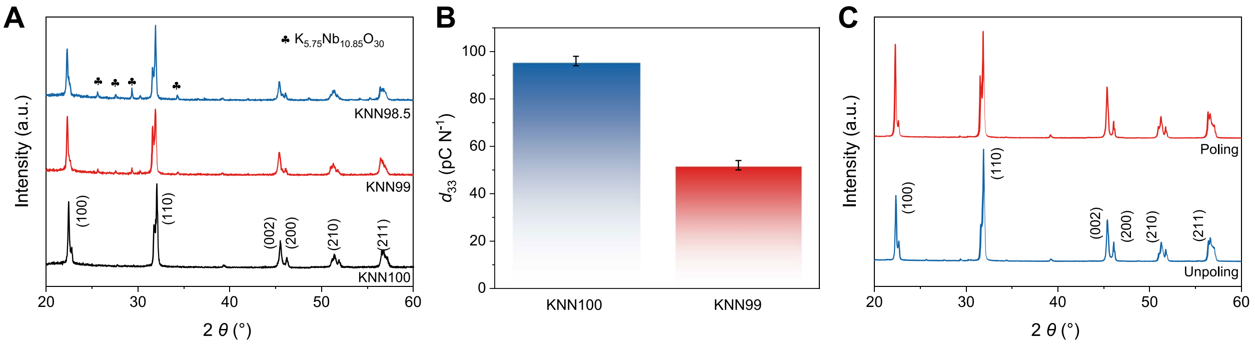


**Fig. S14. XRD patterns and piezoelectric coefficients of KNN ceramics. (A)** XRD patterns of KNN98.5, KNN99, and KNN100 ceramics. (**B**) piezoelectric coefficient $d_{33}$ of KNN99 and KNN100 ceramics. **(C)** XRD patterns of KNN99 ceramics before and after poling. After poling, the intensity of (200) peak decreases, indicating some domains have been switched.

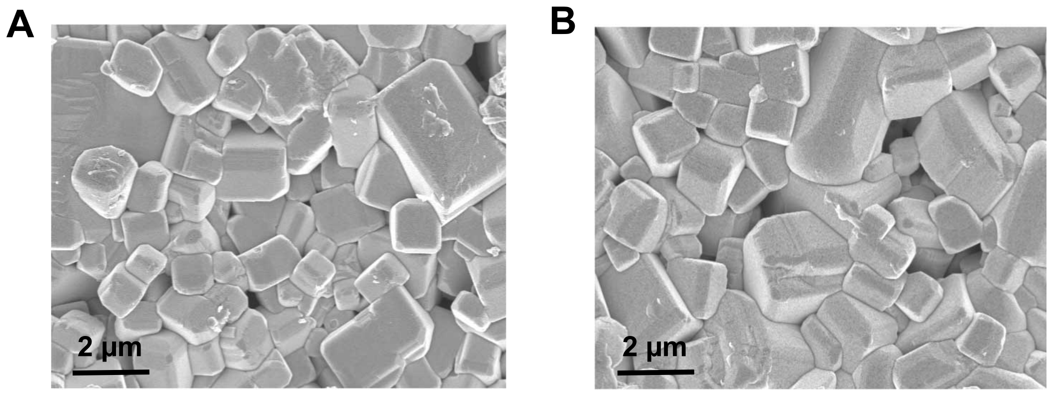


**Fig. S15. Microscopic morphology of KNN ceramics.** SEM images of (**A**) KNN100 and (**B**) KNN99 ceramics.

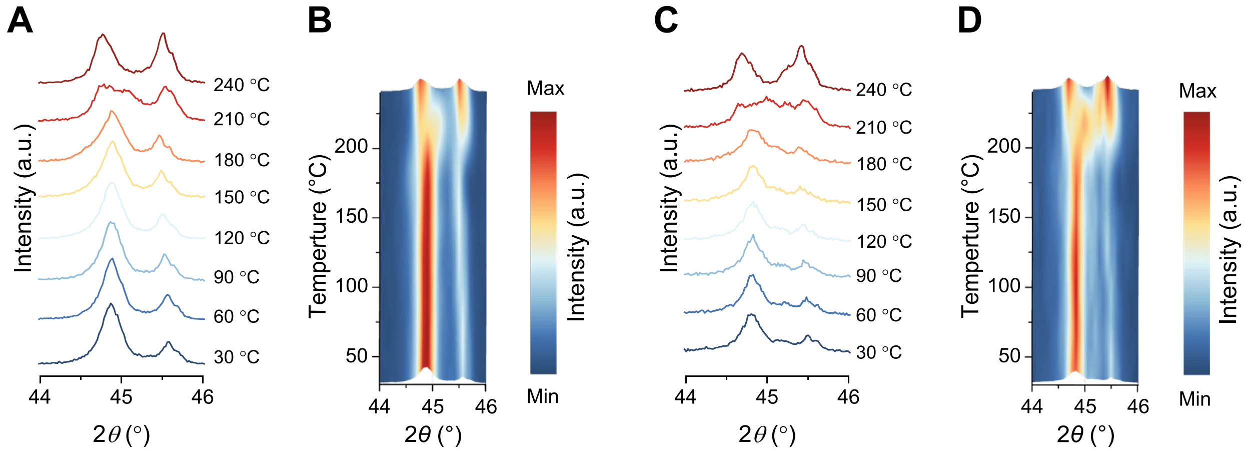


**Fig. S16. In-situ temperature-dependent XRD.** {200} diffraction peaks from 30 °C to 240 °C: (**A**, **B**) KNN99 ceramics and (**C**, **D**) KNN100 ceramics.

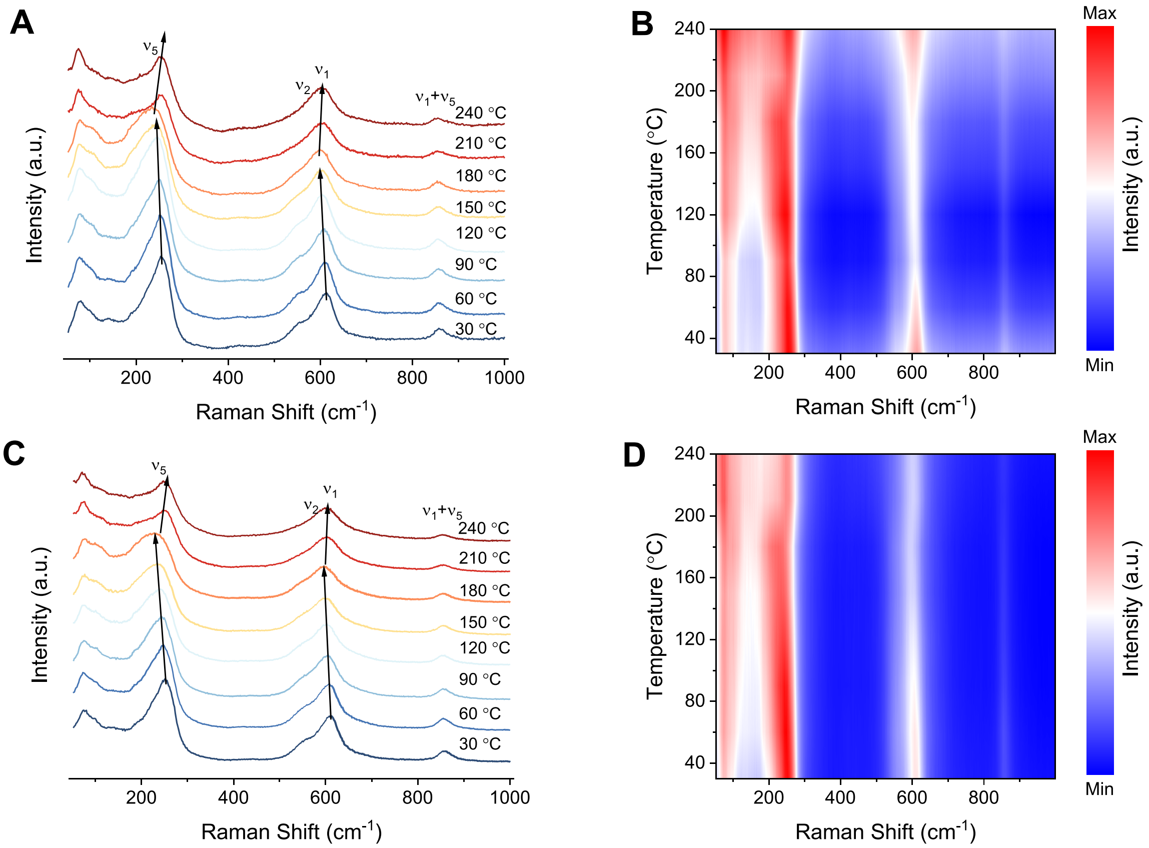


**Fig. S17. In-situ temperature-dependent Raman spectra.** Raman spectra of KNN99 and KNN100 ceramics from 30 °C to 240 °C: (**A**, **B**) KNN99 ceramics and (**C**, **D**) KNN100 ceramics.

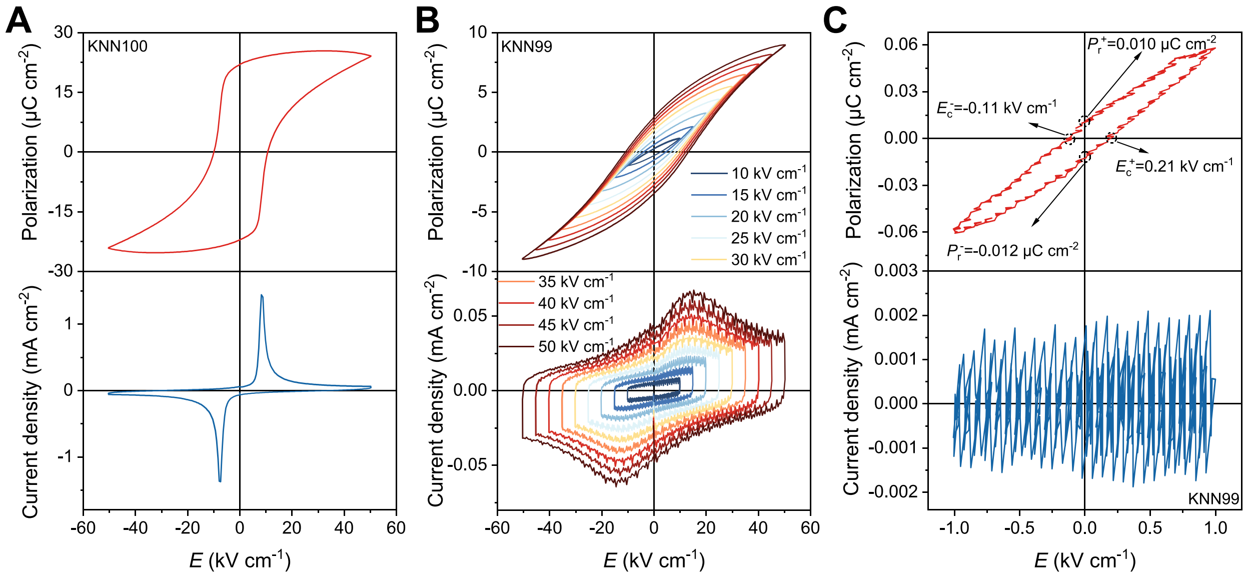


**Fig. S18. Ferroelectric properties of KNN ceramics.** Polarization-electric field (*P*-*E*) loops and current density-electric field (*J*-*E*) curves of (**A**) KNN100 and (**B**) KNN99 ceramics. (**C**) *P*-*E* loop and *J*-*E* curve of KNN99 ceramics under a 1 kV $cm^{-1}$ electric field.

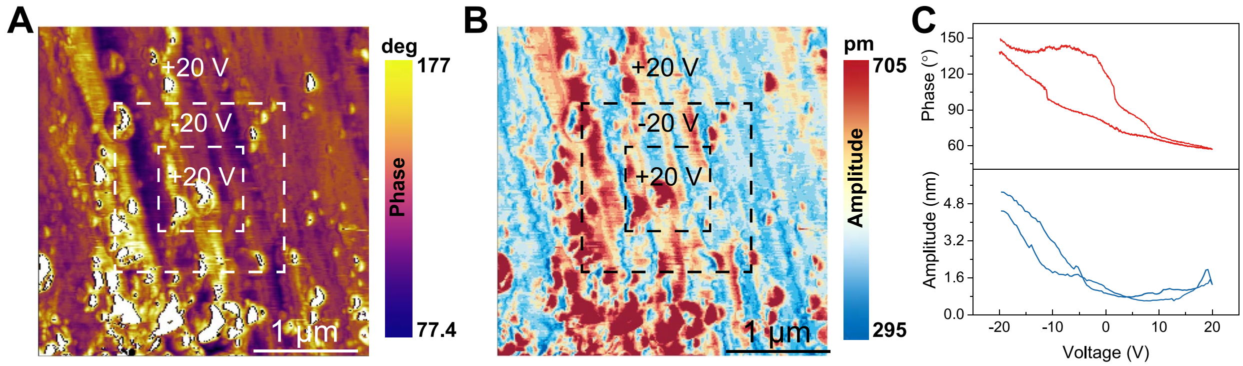


**Fig. S19. PFM characterization of KNN99 ceramics after writing domain measurement.** (**A**) Phase and (**B**) amplitude signals for KNN99 ceramics, respectively. (**C**) Microscopic piezoelectric response phase and amplitude curves under ±20 V voltage loops.

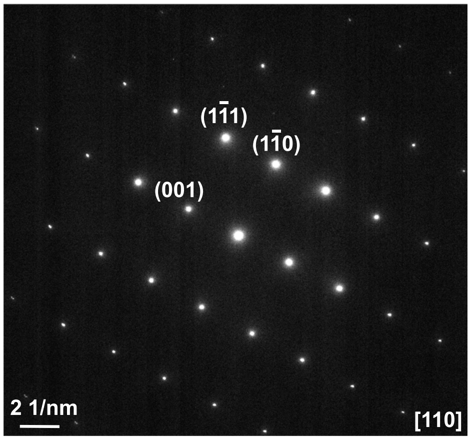


**Fig. S20. Selected area electron diffraction (SAED) pattern of KNN99 ceramics along the [110] zone axis.**

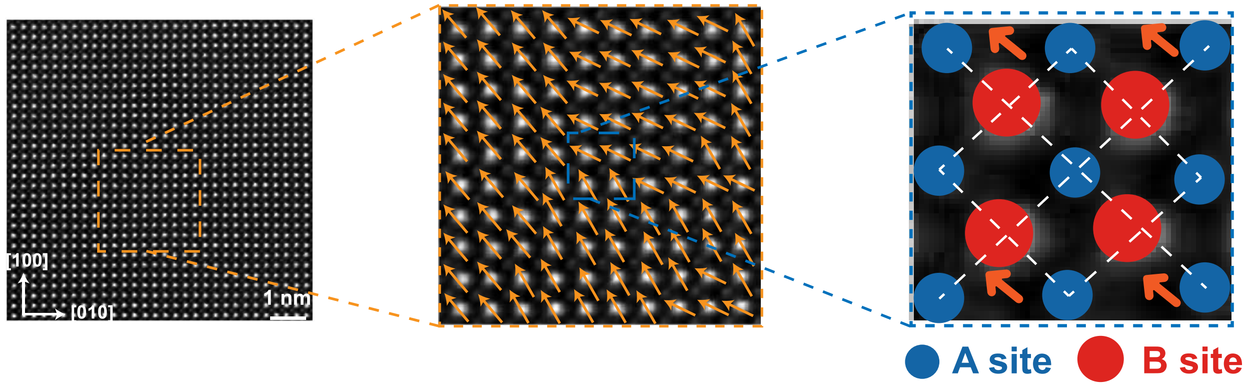


**Fig. S21. Microstructure analysis at the atomic level for KNN99 ceramics.** B-site atom displacement vector in the HAADF image. The orange vectors represent the direction of the B-site atom shift.

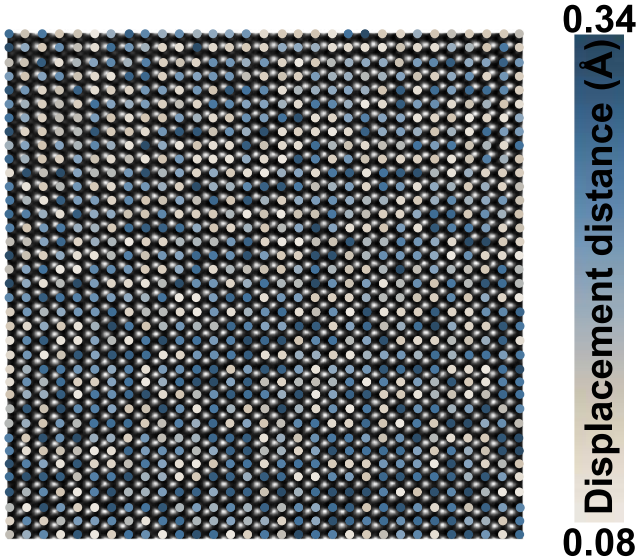


**Fig. S22. A-site cation displacement distance of KNN99 ceramics.**

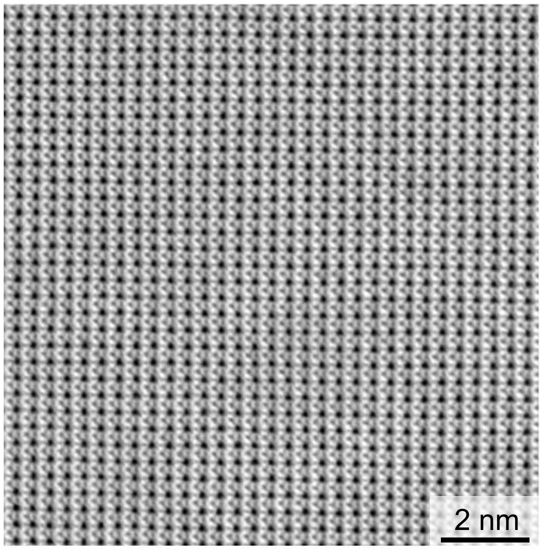

**Fig. S23. ABF image of KNN99 ceramics.**

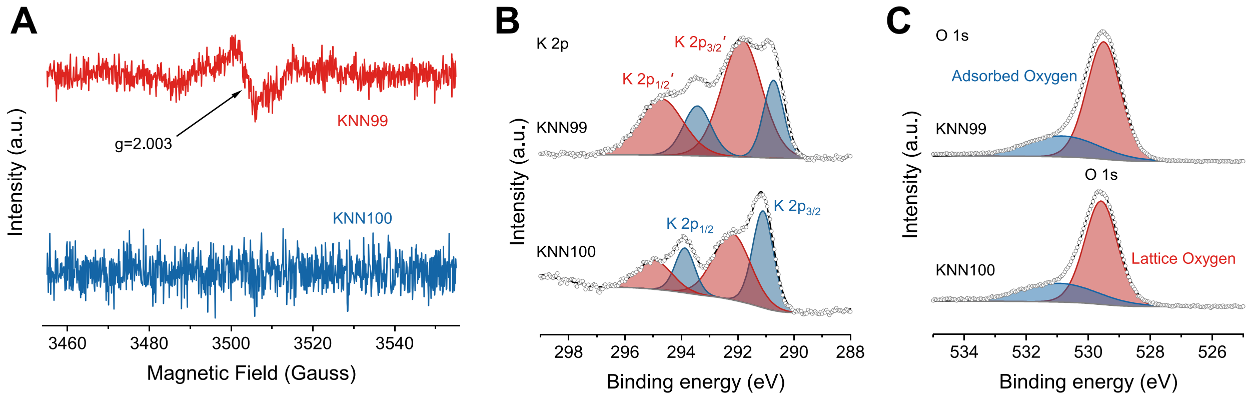


**Fig. S24. Defect characterization of KNN99 and KNN100 ceramics.** (**A**) Electron paramagnetic resonance (EPR). Oxygen vacancy signals occur in KNN99 ceramics (g = 2.003). X-ray photoelectron spectroscopy (XPS) of (**B**) K 2p and (**C**) O 1s. The increase of K $2p_{3/2}$' and K $2p_{1/2}$' peak intensity indicates the increase of potassium vacancies(*40*). The shoulder peak beside the main peak of O 1s is widely believed to correspond to the adsorbed oxygen signal due to oxygen vacancies. The introduction of oxygen vacancies increases the proportion of adsorbed oxygen(*41*).

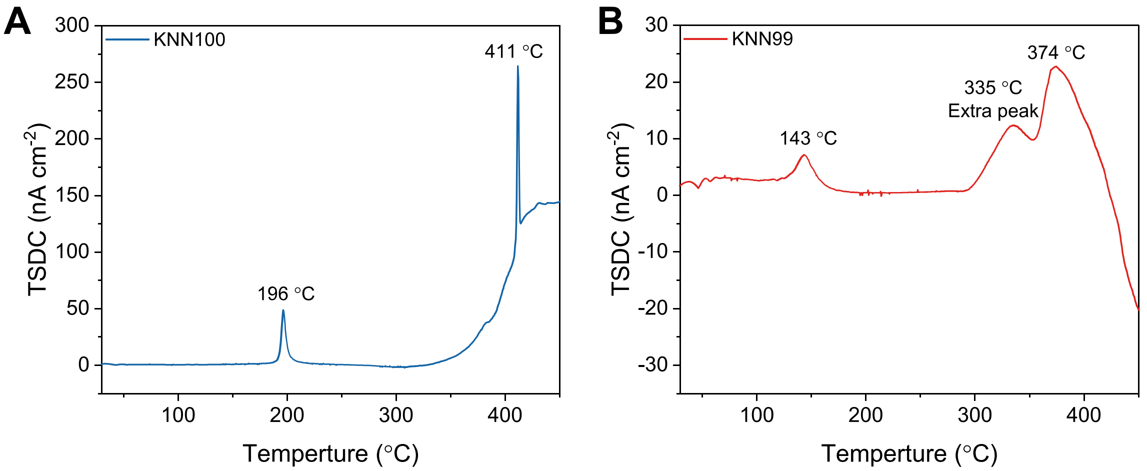


**Fig. S25. Comparison of TSDC spectra of (A) KNN100 and (B) KNN99 ceramics.**

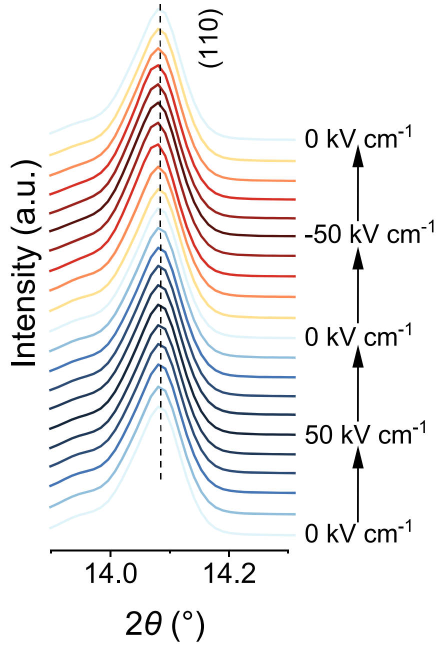


**Fig. S26. In-situ SXRD of {110} diffraction peak for KNN99 ceramics.**

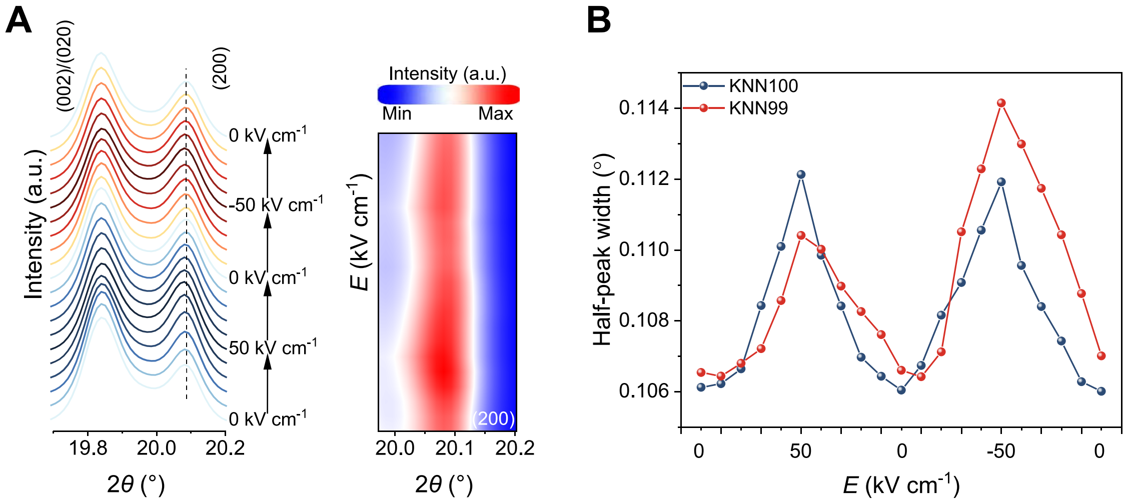


**Fig. S27. In-situ SXRD results of KNN ceramics. (A)** In-situ SXRD of (002)/(020) and (200) peaks for KNN100 ceramics. **(B)** Half-peak widths of the (002) diffraction peak of KNN100 and KNN99 ceramics under different electric fields.

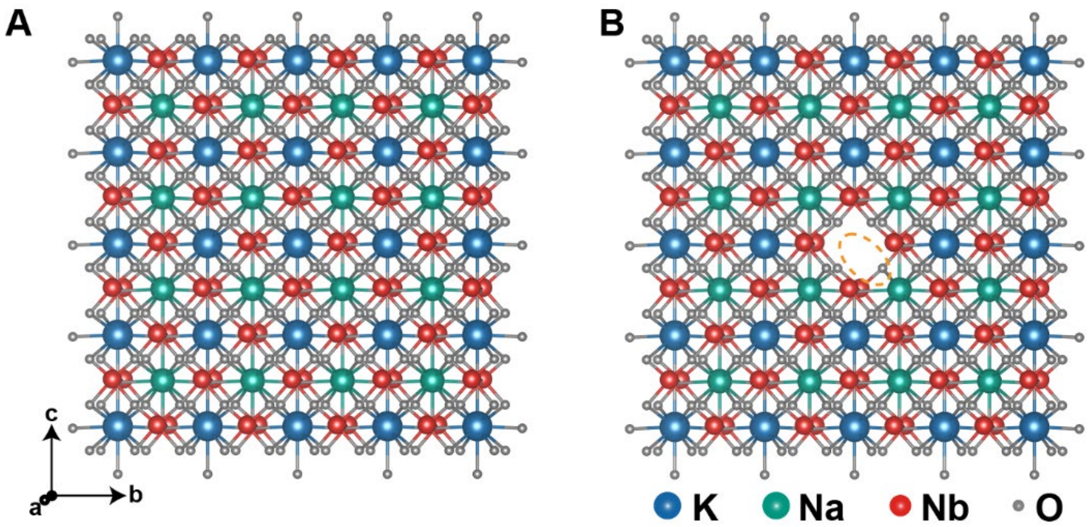


**Fig. S28. Crystal structure models of KNN ceramics** (**A**) without a defect dipole and (**B**) with a defect dipole.

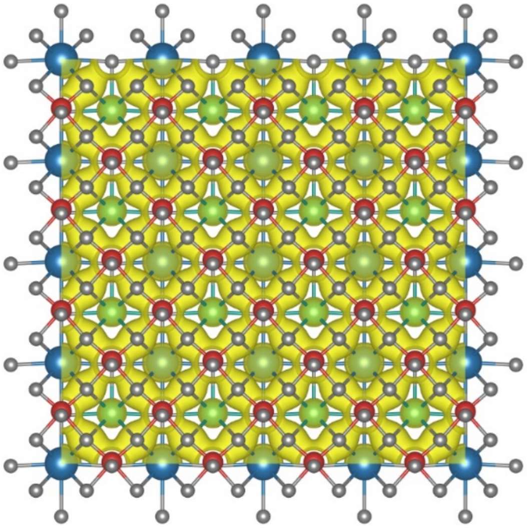

**Fig. S29. Charge density of KNN ceramics without a defect dipole.** The absence of defect dipoles does not cause an uneven distribution of local charge density.

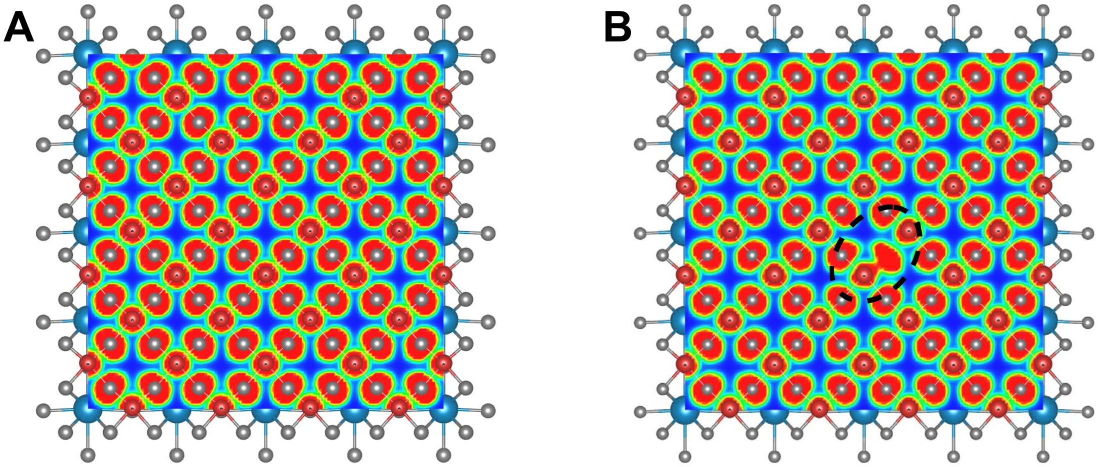


**Fig. S30. Electronic localization function (ELF) analysis of the models:** (**A**) without and (**B**) with a defect dipole.

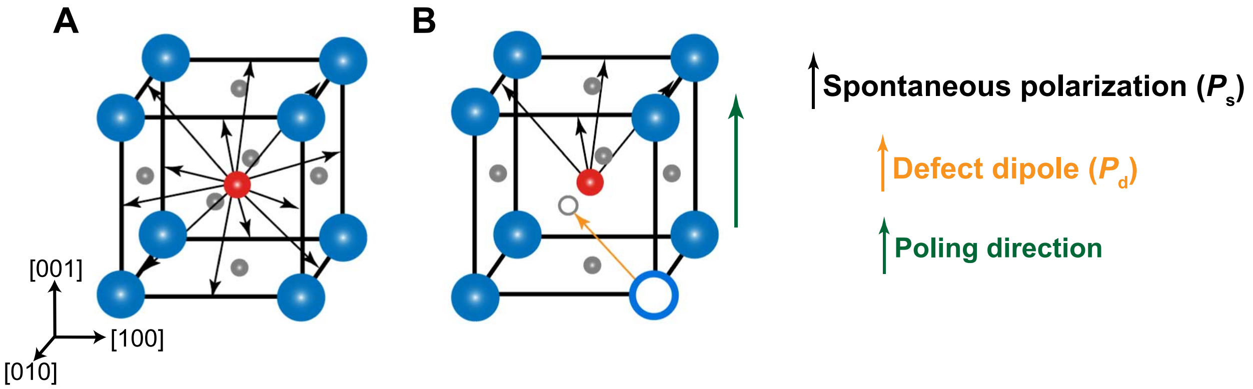


**Fig. S31. Orientation relationship between defect dipole and spontaneous polarization in polycrystalline ceramics.** (**A**) Schematic of the 12 possible <110>-orientations of spontaneous polarizations in one unit cell for pure KNN ceramics. (**B**) A defect dipole is parallel to one of the <110>-orientations after a poling process.

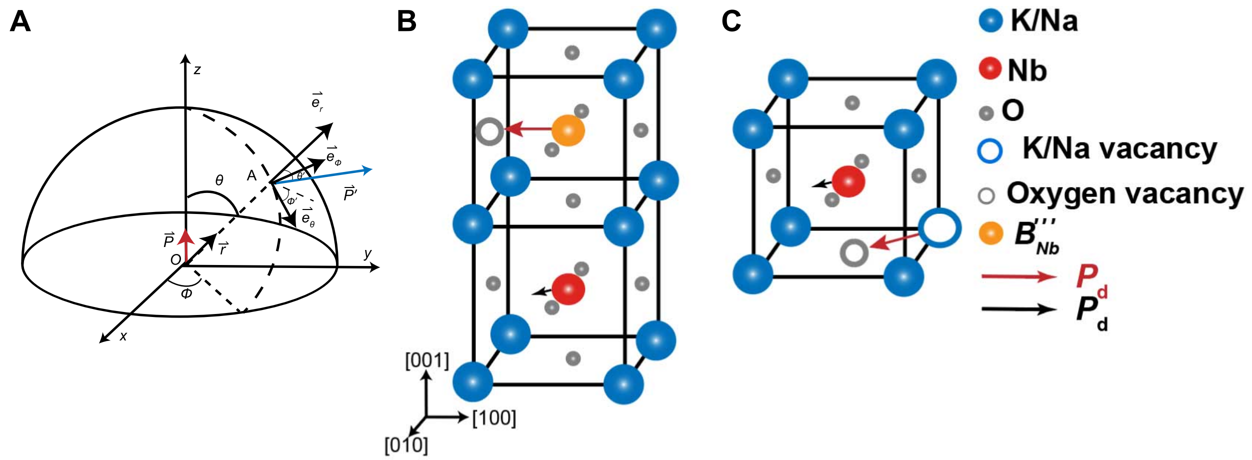


**Fig. S32. Electrostatic energies between defect dipole and ferroelectric spontaneous polarization.** **(A)** Spatial position relationship between two dipoles. **(B)** Schematic diagram of the position and orientation of $(B_{Nb}''' - V_O^{\cdot\cdot})$ defect dipole and the nearest spontaneous polarization. **(C)** Schematic diagram of the position and orientation of $(V_A' - V_O^{\cdot\cdot})$ defect dipole and the nearest spontaneous polarization.

**Note S1** Orientation relationship between defect dipole and spontaneous polarization in polycrystalline ceramics

As illustrated in Fig. S31A, there are 12 possible <110>-orientations of the spontaneous polarizations in one unit cell for KNN ceramics, which possess the orthorhombic phase. Given that the samples are all polycrystalline, the spontaneous polarizations tend to align close to the poling direction while still maintaining the <110>-orientation, i.e., [101], [011], $[0\bar{1}1]$, and $[\bar{1}01]$, as shown in Fig. S31B, representing the degenerate states of the orthorhombic phase. Consequently, the $(V_{\mathrm{A}}' - V_{\mathrm{O}}^{\cdot\cdot})$ defect dipoles are able to establish and align themselves along one of the <110>-orientations due to the electrostatic force during the poling process, which is also in proximity to the poling direction (Fig. S31B). Therefore, in KNN polycrystalline ceramics, while the directions of the <110>-oriented defect dipoles may not be identical, they are all in degenerate states and closely aligned with the poling direction.

**Note S2** Calculation of the electrostatic energy between two dipoles.

As shown in Fig. S32A, Let an electric dipole with dipole moment $\vec{p}$ be located at the origin of the coordinates. The potential of this electric dipole at field point $p(\vec{r})$ is

$$\varphi = \frac{\vec{p}\cdot\vec{r}}{4\pi\varepsilon_0 r^3} \tag{S1}$$

where $\vec{p}$ is dipole moment, $\vec{r}$ is the digit vector of this dipole, $\varepsilon_0$ is the vacuum dielectric constant ~8.85 × $10^{-12}$ F $m^{-1}$, $r$ is the distance between the dipole and the field point $p(\vec{r})$.

For convenience of calculation, make the direction of dipole moment $\vec{p}$ along the z-axis. In the spherical coordinate system, its potential at any point $p(\vec{r})$ in space is

$$\varphi = \frac{\vec{p}\cdot\vec{e}_r}{4\pi\varepsilon_0 r^2} = \frac{p}{4\pi\varepsilon_0 r^2}\cos\,\theta \tag{S2}$$

the electric field intensity is

$$\begin{aligned}\vec{E} &= -\nabla\varphi = -\left(\frac{\partial}{\partial r}\vec{e}_r + \frac{1}{r}\cdot\frac{\partial}{\partial\theta}\vec{e}_\theta + \frac{1}{r\sin\,\theta}\cdot\frac{\partial}{\partial\phi}\vec{e}_\phi\right)\frac{p}{4\pi\varepsilon_0 r^2}\cos\,\theta \\ &= \frac{p}{4\pi\varepsilon_0 r^3}(2\cos\,\theta\vec{e}_r + \sin\,\vec{e}_\theta)\end{aligned} \tag{S3}$$

where $\theta$ is the angle between the digit vector of point $p(\vec{r})$ and the z-axis, $\phi$ is the angle between the projection of the digit vector $\vec{r}$ on the *xoy* plane and the *x*-axis, $\vec{e}_r$, $\vec{e}_\theta$, $\vec{e}_\phi$ are the unit vectors of point $p$.

Let there be another electric dipole at the field point *A* with dipole moment of $\vec{p}'$. Let the angle between the direction of the dipole moment $\vec{p}'$ and the unit vector $\vec{e}_r$ at point *A* be $\theta'$. The angle between the projection of $\vec{p}'$ on the $\vec{e}_\theta p\vec{e}_\phi$ plane and $\vec{e}_\theta$ is $\phi'$. Then the component equation of $\vec{p}'$ is

$$\begin{aligned}\vec{p}' = p'(&\cos\,\theta'\vec{e}_r + \sin\,\theta'\cos\,\phi'\vec{e}_\theta \\ &+\sin\,\theta'\sin\,\phi'\vec{e}_\phi)\end{aligned} \tag{S4}$$

According to the equation for the field energy of an electric dipole in an electrostatic field

$$W = -\vec{p}'\cdot\vec{E} \tag{S5}$$

Bringing the equation (S3) and (S4) to equation (S5):

$$W = -\frac{pp'}{4\pi\varepsilon_0 r^3}(2\cos\,\theta_{\cos}\theta' + \sin\,\theta_{\sin}\theta'\cos\,\phi') \tag{S6}$$

*The electrostatic energy between spontaneous polarization dipole and [001]-direction defect dipole*

We discuss the interaction energy between the defect dipole in the [001]-direction and the spontaneous polarization dipole by means of a $(B_{Nb}''' - V_O^{\cdot\cdot})$ defect dipole formed by acceptor-doped

at the B site. Since the $Nb^{5+}$ in the lattice has been replaced, we consider a $(B_{Nb}''' - V_O^{\cdot\cdot})$ defect dipole in a neighboring cell with a close orientation (Fig. S32B). For convenience of calculation, the unit cell is treated as a cubic. So $\theta$ = 45°, $\theta'$ = $\phi'$ = 0°, the interaction energy between spontaneous polarization dipole and [001]-direction defect dipole is

$$W = -\sqrt{2}\frac{pp'}{4\pi\varepsilon_0 r^3} \quad \text{(S7)}$$

*The electrostatic energy between spontaneous polarization dipole and [110]-direction defect dipole*

For the [110]-defect dipole $(V_A' - V_O^{\cdot\cdot})$, since the defect dipole direction is parallel to the spontaneous polarization direction (Fig. S32C), the $\theta$ = $\theta'$ = $\phi'$ = 45°, the interaction energy between spontaneous polarization dipole and [110]-direction defect dipole is

$$W = -\frac{4+\sqrt{2}}{4}\cdot\frac{pp'}{4\pi\varepsilon_0 r^3} \quad \text{(S8)}$$

The length of the crystalline axis of the unit cell is considered to be 1, so the $r_{[001]}$ = 1, and the $r_{[110]} = \frac{\sqrt{3}}{2}$

The electric moment $\mu d$ of defect dipole could be calculated as follow:

$$\mu_d = ql \quad \text{(S9)}$$

So $\frac{\mu_{d[110]}}{\mu_{d[001]}} = \sqrt{2}$

Finally, equation (S7) and equation (S8) make a ratio

$\frac{W[110]}{W[001]} \approx 2.95$

**Note S3** The relationship between thickness effect and defect dipole.

To gain a deeper understanding of the thickness dependence of electrostrain, particularly dominated by defect dipoles in KNN99 ceramics, we conducted finite element simulations using COMSOL Multiphysics. The models were constructed using two piezoelectric materials with varying stiffness: the harder material (PZT-8) served as the matrix (7×7 per layer) to simulate ferroelectric domains, while the softer material (PZT-5J) represented defect dipoles. Building upon the mechanism proposed in our previous work(*8*), where aligned defect dipoles drive lattice stretching or contraction in neighboring crystalline lattices, it is expected that defect dipoles should exhibit lower stiffness and a more robust electromechanical response under external electric fields. Fig. S4 displays the models used in the simulations depicting the thickness dependence of electrostrain with different defect dipole concentrations, and the corresponding simulation results are summarized in Fig. S5. The results in Fig. S4 demonstrate a decreasing trend in strain with increasing thickness, aligning well with the experimental findings in Extended Data Figs. 2a and 2b. Despite a constant concentration of defect dipoles, an increase in sample thickness intensifies the clamping effect on the defect dipoles by the ferroelectric domains with higher stiffness surrounding them. Consequently, the strain values of KNN99 ceramics exhibit a rapid increase with sample thinning. Furthermore, Fig. S5 indicates that the influence of thickness is more pronounced in ceramics with lower defect dipole concentration, suggesting the potential for designing higher defect dipole contents to achieve thickness-independent electrostrain in future endeavors.

**Table S1.** Comparison of ion displacements of KNN ceramics without and with defect dipole.

| Without defect dipole | | | | With defect dipole | | | |
|---|---|---|---|---|---|---|---|
| Atom (O) | Displacement (Å) | Atom (Nb) | Displacement (Å) | Atom (O) | Displacement (Å) | Atom (Nb) | Displacement (Å) |
| O1 | 0.022 | Nb1 | 0.027 | O1 | 0.103 | Nb1 | 0.175 |
| O2 | 0.031 | Nb2 | 0.025 | O2 | 0.316 | Nb2 | 0.182 |
| O3 | 0.037 | Nb3 | 0.031 | O3 | 0.143 | Nb3 | 0.184 |
| O4 | 0.025 | Nb4 | 0.032 | O4 | 0.157 | Nb4 | 0.186 |
| O5 | 0.014 | Nb5 | 0.016 | O5 | 0.294 | Nb5 | 0.179 |
| O6 | 0.016 | Nb6 | 0.018 | O6 | 0.289 | Nb6 | 0.181 |
| O7 | 0.018 | Nb7 | 0.024 | O7 | 0.171 | Nb7 | 0.196 |
| O8 | 0.017 | Nb8 | 0.026 | O8 | 0.172 | Nb8 | 0.202 |
| O9 | 0.033 | Average | 0.0245 | O9 | 0.232 | Average | 0.186 |
| O10 | 0.032 | Na1 | 0.017 | O10 | 0.232 | Na1 | 0.181 |
| O11 | 0.037 | Na2 | 0.011 | O11 | 0.133 | Na2 | 0.216 |
| O12 | 0.034 | Na3 | 0.014 | O12 | - | Na3 | 0.202 |
| Average | 0.026 | Na4 | 0.014 | Average | 0.204 | Na4 | 0.164 |
| | | Average | 0.014 | | | Average | 0.191 |

**Table S2.** Effective piezoelectric strain coefficient $d_{33}^*$ and hysteresis of KNN99 ceramics under different electric fields.

| $E$ (kV cm$^{-1}$) | | 10 | 15 | 20 | 25 | 30 | 35 | 40 | 45 | 50 |
|---|---|---|---|---|---|---|---|---|---|---|
| Unipolar | $S_{max}/E_{max}$ (pm V$^{-1}$) | 2800 | 3267 | 3500 | 3720 | 3867 | 3971 | 4000 | 4111 | 4200 |
| | Hysteresis (%) | 18.6 | 20.4 | 21.3 | 23.8 | 23.9 | 24.6 | 23.6 | 23.7 | 23.2 |
| Bipolar | $S_{max}/E_{max}$ (pm V$^{-1}$) | 2900 | 3933 | 4650 | 5400 | 5833 | 6029 | 6125 | 6156 | 6200 |
| | Hysteresis (%) | 43.8 | 43.1 | 46.6 | 46 | 45.9 | 45.1 | 42.3 | 41.8 | 29.3 |

**Table S3.** Effective piezoelectric strain coefficient $d_{33}^*$ and hysteresis of KNN99 ceramics under different temperatures.

| $T$ (°C) | | 30 | 60 | 90 | 120 | 150 | 180 | 210 | 240 |
|---|---|---|---|---|---|---|---|---|---|
| Unipolar | $S_{max}/E_{max}$ (pm $V^{-1}$) | 4020 | 4280 | 4380 | 4580 | 5800 | 6160 | 8800 | 7520 |
| | Hysteresis (%) | 25.6 | 26.4 | 25.2 | 23.9 | 23.2 | 31 | 32.3 | 40.2 |
| Bipolar | $S_{max}/E_{max}$ (pm $V^{-1}$) | 6000 | 6020 | 6700 | 7700 | 8860 | 9480 | 10600 | 9580 |
| | Hysteresis (%) | 37.2 | 35.5 | 31.4 | 28.2 | 25.4 | 24.6 | 26.2 | 36.3 |

**Table S4.** Electrostrain performance of state-of-the-art piezoelectric materials with their detailed information.

| Materials | $S_{max}$ (%) | $S_{max}/E_{max}$ (pm $V^{-1}$) | Structure | Lead-free | Journal/Year | Ref. |
|---|---|---|---|---|---|---|
| KNN-Li/Ta | 0.15 | 750 | Textured ceramic | ✓ | Nature/2004 | 4 |
| KNN-Cu | 0.5 | 1500 | Polycrystalline ceramic | ✓ | Acta Mater/2020 | 8 |
| BNT-Sr/Nb | 1.6 | 1600 | Textured ceramic | ✓ | Adv Mater/2023 | 9 |
| BNT (220 °C) | 2.3 | 2555 | Polycrystalline ceramic | ✓ | Nat Commun/2022 | 10 |
| BNT | 1.1 | 1100 | Polycrystalline ceramic | ✓ | Sci Adv/2023 | 12 |
| KNN-Sr | 0.7 | 1400 | Polycrystalline ceramic | ✓ | Science/2022 | 13 |
| KNN-Sr (160 °C) | 1.2/1.65 | 2400/3300 | | | | |
| KNN-Sr/Ni | 0.8 | 1600 | Textured ceramic | ✓ | Adv Funct Mater/2023 | 14 |
| PMN-PT | 0.3 | 3000 | Single crystal | × | Science/2019 | 15 |
| KNN-Li | 5 | 6250 | Polycrystalline ceramic | ✓ | J Appl Phys/2023 | 19 |
| PMN-PT | 1.7 | 1417 | Single crystal | × | J Appl Phys/1997 | 22 |
| BLF-PT | 1.3 | 1625 | Polycrystalline ceramic | × | Nat Mater/2018 | 23 |
| BNT-BT-KNN | 0.9 | 2571 | Single crystal | ✓ | Adv Electron Mater/2019 | 25 |
| PZT | 0.33 | 550 | Polycrystalline ceramic | × | Phys Rev Lett/2016 | 26 |
| BCZT | 0.24 | 1200 | Textured ceramic | ✓ | Acs Appl Mater Inter/2017 | 27 |
| BNKT-BT | 0.41 | 1025 | Textured ceramic | ✓ | Mater Design/2018 | 28 |
| BNT-Sr/Li | 0.74 | 1057 | Polycrystalline ceramic | ✓ | Nano Energy/2020 | 29 |

| | | | | | | |
|---|---|---|---|---|---|---|
| BF-BT-BZT | 0.97 | 1213 | Polycrystalline ceramic | ✓ | Mater Today Chem/2022 | 30 |
| PIN-PSN-PT | 0.6 | 2000 | Textured ceramic | × | Nat Commun/2021 | 31 |
| BNKT-BT | 0.87 | 2175 | Single crystal | ✓ | Appl Phys Lett/2008 | 32 |
| BNT-BT-KNN | 0.83 | 2976 | Single crystal | ✓ | Appl Phys Lett/2016 | 33 |
| KNN99 | 3.1 | 6200 | Polycrystalline ceramic | ✓ | This Work | |
| KNN99 (210 °C) | 5.3 | 10600 | | | | |

**REFERENCES AND NOTES**


1. J. F. Scott, Applications of modern ferroelectrics. *Science* **315**, 954–959 (2007).

2. F. Li, L. Jin, Z. Xu, S. J. Zhang, Electrostrictive effect in ferroelectrics: An alternative approach to improve piezoelectricity. *Appl. Phys. Rev.* **1**, 011103 (2014).

3. G. Viola, Y. Tian, C. Y. Yu, Y. Q. Tan, V. Koval, X. Y. Wei, K. L. Choy, H. X. Yan, Electric field-induced transformations in bismuth sodium titanate-based materials. *Prog. Mater. Sci.* **122**, 100837 (2021).

4. Y. Saito, H. Takao, T. Tani, T. Nonoyama, K. Takatori, T. Homma, T. Nagaya, M. Nakamura, Lead-free piezoceramics. *Nature* **432**, 84–87 (2004).

5. J. G. Hao, W. Li, J. W. Zhai, H. Chen, Progress in high-strain perovskite piezoelectric ceramics. *Mater. Sci. Eng. R Rep.* **135**, 1–57 (2019).

6. Z. H. Zhao, Y. J. Dai, F. Huang, The formation and effect of defect dipoles in lead-free piezoelectric ceramics: A review. *Sustain. Mater. Techno.* **20**, e00092 (2019).

7. X. B. Ren, Large electric-field-induced strain in ferroelectric crystals by point-defect-mediated reversible domain switching. *Nat. Mater.* **3**, 91–94 (2004).

8. Z. H. Zhao, Y. K. Lv, Y. J. Dai, S. J. Zhang, Ultrahigh electro-strain in acceptor-doped KNN lead-free piezoelectric ceramics via defect engineering. *Acta Mater.* **200**, 35–41 (2020).

9. L. X. Lai, B. Li, S. Tian, Z. H. Zhao, S. J. Zhang, Y. J. Dai, Giant electrostrain in lead-free textured piezoceramics by defect dipole design. *Adv. Mater.* **35**, e2300519 (2023).

10. W. Feng, B. C. Luo, S. S. Bian, E. K. Tian, Z. L. Zhang, A. Kursumovic, J. L. MacManus-Driscoll, X. H. Wang, L. T. Li, Heterostrain-enabled ultrahigh electrostrain in lead-free piezoelectric. *Nat. Commun.* **13**, 5086 (2022).

11. Z. H. Zhao, Y. J. Dai, X. L. Li, Z. Zhao, X. W. Zhang, The evolution mechanism of defect dipoles and high strain in $MnO_2$-doped KNN lead-free ceramics. *Appl. Phys. Lett.* **108**, 172906 (2016).

12. H. Luo, H. Liu, H. Huang, Y. Song, M. G. Tucker, Z. Sun, Y. Yao, B. Gao, Y. Ren, M. Tang, H. Qi, S. Deng, S. Zhang, J. Chen, Achieving giant electrostrain of above 1% in (Bi,Na)$TiO_3$-based lead-free piezoelectrics via introducing oxygen-defect composition. *Sci. Adv.* **9**, eade7078 (2023).

13. G. Huangfu, K. Zeng, B. Wang, J. Wang, Z. Fu, F. Xu, S. Zhang, H. Luo, D. Viehland, Y. Guo, Giant electric field–induced strain in lead-free piezoceramics. *Science* **378**, 1125–1130 (2022).

14. B. Q. Wang, G. Huangfu, Z. P. Zheng, Y. P. Guo, Giant electric field-induced strain with high temperature-stability in textured KNN-based piezoceramics for actuator applications. *Adv. Funct. Mater.* **33**, 2214643 (2023).

15. F. Li, M. J. Cabral, B. Xu, Z. X. Cheng, E. C. Dickey, J. M. LeBeau, J. L. Wang, J. Luo, S. Taylor, W. Hackenberger, L. Bellaiche, Z. Xu, L. Q. Chen, T. R. Shrout, S. J. Zhang, Giant piezoelectricity of Sm-doped $Pb(Mg_{1/3}Nb_{2/3})O_3$-$PbTiO_3$ single crystals. *Science* **364**, 264–268 (2019).

16. J. L. Li, W. B. Qu, J. Daniels, H. J. Wu, L. J. Liu, J. Wu, M. W. Wang, S. Checchia, S. Yang, H. B. Lei, R. Lv, Y. Zhang, D. Y. Wang, X. X. Li, X. D. Ding, J. Sun, Z. Xu, Y. F. Chang, S. J. Zhang, F. Li, Lead zirconate titanate ceramics with aligned crystallite grains. *Science* **380**, 87–93 (2023).

17. X. Lv, J. G. Zhu, D. Q. Xiao, X. X. Zhang, J. G. Wu, Emerging new phase boundary in potassium sodium-niobate based ceramics. *Chem. Soc. Rev.* **49**, 671–707 (2020).

18. X. B. Ren, K. Otsuka, Universal symmetry property of point defects in crystals. *Phys. Rev. Lett.* **85**, 1016–1019 (2000).

19. G. D. Adhikary, D. N. Singh, G. A. Tina, G. J. Muleta, R. Ranjan, Ultrahigh electrostrain > 1% in lead-free piezoceramics: Role of disk dimension. *J. Appl. Phys.* **134**, 054101 (2023).

20. D. S. Park, M. Hadad, L. M. Riemer, R. Ignatans, D. Spirito, Esposito, Tileli, N. Gauquelin, D. Chezganov, D. Jannis, J. Verbeeck, S. Gorfman, N. Pryds, P. Muralt, D. Damjanovic, Induced giant piezoelectricity in centrosymmetric oxides. *Science* **375**, 653–657 (2022).

21. D. Wang, Y. Fotinich, G. P. Carman, Influence of temperature on the electromechanical and fatigue behavior of piezoelectric ceramics. *J. Appl. Phys.* **83**, 5342–5350 (1998).

22. S.-E. Park, T. R. Shrout, Ultrahigh strain and piezoelectric behavior in relaxor based ferroelectric single crystals. *J. Appl. Phys.* **82**, 1804–1811 (1997).

23. B. Narayan, J. S. Malhotra, R. Pandey, K. Yaddanapudi, P. Nukala, B. Dkhil, A. Senyshyn, R. Ranjan, Electrostrain in excess of 1% in polycrystalline piezoelectrics. *Nat. Mater.* **17**, 427–431 (2018).

24. X. M. Liu, X. L. Tan, Giant strains in non-textured $(Bi_{1/2}Na_{1/2})TiO_3$-based lead-free ceramics. *Adv. Mater.* **28**, 574–578 (2016).

25. Y. Wang, C. Luo, S. Wang, C. Chen, G. Yuan, H. Luo, D. Viehland, Large piezoelectricity in ternary lead-free single crystals. *Adv. Electron. Mater.* **6**, 1900949 (2020).

26. L. L. Fan, J. Chen, Y. Ren, Z. Pan, L. X. Zhang, X. R. Xing, Unique piezoelectric properties of the monoclinic phase in $Pb(Zr,Ti)O_3$ ceramics: Large lattice strain and negligible domain switching. *Phys. Rev. Lett.* **116**, 027601 (2016).

27. Y. C. Liu, Y. F. Chang, F. Li, B. Yang, Y. Sun, J. Wu, S. T. Zhang, R. X. Wang, W. W. Cao, Exceptionally high piezoelectric coefficient and low strain hysteresis in grain-oriented $(Ba, Ca)(Ti, Zr)O_3$ through integrating crystallographic texture and domain engineering. *Acs. Appl. Mater. Interfaces* **9**, 29863–29871 (2017).

28. Z.-H. Zhao, M.-Y. Ye, H.-M. Ji, X.-L. Li, X. Zhang, Y. Dai, Enhanced piezoelectric properties and strain response in <001> textured BNT-BKT-BT ceramics. *Mater. Design* **137**, 184–191 (2018).

29. J. Y. Wu, H. B. Zhang, C. H. Huang, C. W. Tseng, N. Meng, V. Koval, Y. C. Chou, Z. Zhang, H. X. Yan, Ultrahigh field-induced strain in lead-free ceramics. *Nano Energy* **76**, 105037 (2020).

30. W. Li, C. Zhou, J. Wang, C. Yuan, J. Xu, Q. Li, G. Chen, J. Zhao, G. Rao, Giant electro-strain nearly 1% in $BiFeO_3$-based lead-free piezoelectric ceramics through coupling morphotropic phase boundary with defect engineering. *Mater. Today. Chem.* **26**, 101237 (2022).

31. S. Yang, J. L. Li, Y. Liu, M. W. Wang, L. Qiao, X. Y. Gao, Y. F. Chang, H. L. Du, Z. Xu, S. J. Zhang, F. Li, Textured ferroelectric ceramics with high electromechanical coupling factors over a broad temperature range. *Nat. Commun.* **12**, 1414 (2021).

32. S. Teranishi, M. Suzuki, Y. Noguchi, M. Miyayama, C. Moriyoshi, Y. Kuroiwa, K. Tawa, S. Mori, Giant strain in lead-free $(Bi_{0.5}Na_{0.5})TiO_3$-based single crystals. *Appl. Phys. Lett.* **92**, 182905 (2008).

33. C. Chen, X. Y. Zhao, Y. J. Wang, H. W. Zhang, H. Deng, X. B. Li, X. G. Jiang, X. P. Jiang, H. S. Luo, Giant strain and electric-field-induced phase transition in lead-free $(Na_{0.5}Bi_{0.5})TiO_3$-$BaTiO_{3}$-$(K_{0.5}Na_{0.5})NbO_3$ single crystal. *Appl. Phys. Lett.* **108**, 022903 (2016).

34. B. Wang, G. Huangfu, J. Wang, S. Zhang, Y. Guo, Lead-free piezoceramic macro-fiber composite actuators toward active vibration control systems. *J. Materiomics* **10**, 78–85 (2024).

35. H.-C. Thong, A. Payne, J.-W. Li, J. L. Jones, K. Wang, The origin of chemical inhomogeneity in lead-free potassium sodium niobate ceramic: Competitive chemical reaction during solid-state synthesis. *Acta Mater.* **211**, 116833 (2021).

36. G. Kresse, J. Hafner, Ab initio molecular dynamics for liquid metals. *Phys. Rev. B* **47**, 558–561 (1993).

37. G. Kresse, J. Furthmüller, Efficient iterative schemes forab initiototal-energy calculations using a plane-wave basis set. *Phys. Rev. B* **54**, 11169–11186 (1996).

38. J. P. Perdew, K. Burke, M. Ernzerhof, Generalized gradient approximation made simple. *Phys. Rev. Lett.* **77**, 3865–3868 (1996).

39. J. D. Pack, H. J. Monkhorst, "Special points for Brillouin-zone integrations"—A reply. *Phys. Rev. B* **16**, 1748–1749 (1977).

40. L. Y. Wang, K. Yao, P. C. Goh, W. Ren, Volatilization of alkali ions and effects of molecular weight of polyvinylpyrrolidone introduced in solution-derived ferroelectric $K_{0.5}Na_{0.5}NbO_3$ films. *J. Mater. Res.* **24**, 3516–3522 (2009).

41. H. J. Luo, H. Liu, S. Q. Deng, S. X. Hu, L. Wang, B. T. Gao, S. D. Sun, Y. Ren, L. J. Qiao, J. Chen, Simultaneously enhancing piezoelectric performance and thermal depolarization in lead-free (Bi,Na)$TiO_3$-$BaTiO_3$ via introducing oxygen-defect perovskites. *Acta Mater.* **208**, 116711 (2021).
